# TCMP‐2 affects tomato flowering and interacts with BBX16, a homolog of the arabidopsis B‐box MiP1b

**DOI:** 10.1002/pld3.283

**Published:** 2020-11-07

**Authors:** Barbara Molesini, Valentina Dusi, Federica Pennisi, Gian Pietro Di Sansebastiano, Serena Zanzoni, Anna Manara, Antonella Furini, Flavio Martini, Giuseppe Leonardo Rotino, Tiziana Pandolfini

**Affiliations:** ^1^ Department of Biotechnology University of Verona Verona Italy; ^2^ DiSTeBA Department of Biological and Environmental Sciences and Technologies University of Salento Lecce Italy; ^3^ Centro Piattaforme Tecnologiche University of Verona Verona Italy; ^4^ CREA Research Centre for Genomics and Bioinformatics Lodi Italy

## Abstract

Flowering and fruiting are processes subject to complex control by environmental and endogenous signals. Endogenous signals comprise, besides classical phytohormones, also signaling peptides and miniproteins. Tomato cystine‐knot miniproteins (TCMPs), which belong to a Solanaceous‐specific group of Cys‐rich protein family, have been recently involved in fruit development. *TCMP‐1* and *TCMP‐2* display a highly modulated expression pattern during flower and fruit development. A previous study reported that a change in the ratio of the two TCMPs affects the timing of fruit production. In this work, to investigate TCMP‐2 mode of action, we searched for its interacting partners. One of the interactors identified by a yeast two hybrid screen, was the B‐box domain‐containing protein 16 (*Sl*BBX16), whose closest homolog is the Arabidopsis microProtein 1b implicated in flowering time control. We demonstrated the possibility for the two proteins to interact *in vivo* in tobacco epidermal cells. Arabidopsis plants ectopically overexpressing the TCMP‐2 exhibited an increased level of *FLOWERING LOCUS T* (*FT*) mRNA and anticipated flowering. Similarly, in previously generated transgenic tomato plants with increased *TCMP‐2* expression in flower buds, we observed an augmented expression of *SINGLE‐FLOWER TRUSS* gene, the tomato ortholog of *FT*, whereas the expression of the antiflorigen *SELF‐PRUNING* was unchanged. Consistently, these transgenic plants showed alterations in the flowering pattern, with an accelerated termination of the sympodial units. Overall, our study reveals a novel function for TCMP‐2 as regulatory factor that might integrate, thanks to its capacity to interact with *Sl*BBX16, into the signaling pathways that control flowering, and converge toward florigen regulation.

## 1 INTRODUCTION

Cystine‐knot proteins are characterized by a small size, usually less than 50 amino acids in their mature form, and by the presence of six conserved Cys residues linked by three disulphide bonds in a knotted arrangement (Rees & Lipscomb, 1982). Even if the knot motif is widespread in various species such as fungi, insects, molluscs, and mammals, proteins belonging to the cystine‐knot type display diversified roles, mainly in defence against microorganisms acting as toxins (e.g. ion channel or enzyme inhibitors) and in cell signaling (Craik, 2001; Iyer & Acharya, 2011; Schwarz, 2017; Vitt et al., 2014). Cystine‐knot proteins are also well‐represented in several plant species including medicinal herbs and crops, and most are inhibitors of proteases, namely metallocarboxypeptidases and serine proteases (Daly & Craik, 2011; Molesini et al., 2017). Certain protease inhibitors have a regulatory role preventing uncontrolled proteolysis, while others serve as storage proteins, and others are antifeedant and antimicrobial molecules implicated in plant protection against insects and microorganisms, respectively (Hartl et al., 2010; Norton & Pallaghy, 1998; Quilis et al., 2007; Ryan, 1980). A class of cystine‐knot protease inhibitors restricted to the Solanaceae family was described for the first time in the 1980s (Hass & Hermodson, 1981; Rees & Lipscomb, 1980). This group includes two tomato genes, indicated as tomato cystine‐knot miniproteins 1 and 2 (*TCMP‐1* and *TCMP‐2*), which code for metallocarboxypeptidase inhibitors of 37 and 44 amino acids, respectively. *TCMP‐1* and *TCMP‐2* display a sequential expression pattern, which is highly modulated during flower and fruit development. *TCMP‐1* is expressed at very high level in flower buds before anthesis, then its expression decreases rapidly after anthesis and increases again during fruit development (Cavallini et al., 2011). *TCMP‐1* mRNA level is very low in leaves, although its expression is induced by wounding and elicitors of responses to biotic stress (Díez‐Díaz et al., 2004; Martineau et al., 1991). Recently, *TCMP‐1* has been proved to be also responsive to abiotic stress, such as saline stress and Cd toxicity (Manara et al., 2020). The expression of *TCMP‐2* is apparently absent in leaves, roots, and stems (Pear et al., 1989), is low in flower buds before anthesis, and gradually increases after fertilization reaching a maximum in green and ripe fruits (Cavallini et al., 2011; Treggiari et al., 2015). In fact, the *TCMP‐2* promoter (also named 2A11; X13743; [Pear et al., 1989]) has been successfully used to improve qualitative trait in tomato fruit (Davuluri et al., 2005).

Although the biological activity of metallocarboxypeptidase inhibitors argues in favor of a role for Solanaceous cystine‐knot proteins in plant defence, it has been recently demonstrated that tomato TCMPs are implicated in fruit development (Molesini et al., 2018). Indeed, many lines of evidence indicate that several genes implicated in plant defence play also a role in development regulation (Hartl et al., 2011). In the paper by Molesini et al. (2018), tomato plants were transformed with a chimeric gene containing the entire *TCMP‐1* coding region under the control of the *TCMP‐2* promoter (*pTCMP‐2::TCMP‐1*). As expected by the use of the fruit‐specific *TCMP‐2* promoter, an increased expression of *TCMP‐1* was observed in green and ripe fruits, resulting only in a slight delay in the ripening. Unexpectedly, *pTCMP‐2::TCMP‐1* plants exhibited a marked increase in the expression of *TCMP‐2* before anthesis associated with an anticipated fruit production, that might be due to mechanisms controlling TCMPs homeostasis (Molesini et al., 2018). Thus, it is conceivable that the maintenance of a proper TCMPs ratio is required for regulating fruit set timing.

The mode of action of TCMPs in plants remains largely unveiled also for the absence of homologous genes in other model species, including Arabidopsis. In several cases, Cys‐rich peptides act as signaling molecules in plant development by interfering with receptors or modifying the activity of multimeric complexes (De Coninck & De Smet, 2016; Tavormina et al., 2015).

Here, to gain further insights on the role of TCMP‐2 in reproductive development, we conducted a yeast two‐hybrid (Y2H) screen to discover its potential cellular partners. Among the identified interactors, we found the B‐box domain‐containing protein 16 (*Sl*BBX16). The interaction between TCMP‐2 and *Sl*BBX16 was validated *in vivo* in plant cells by bimolecular fluorescence complementation (BIFC) analysis. We demonstrated that TCMP‐2 is also able to interact with the Arabidopsis closest homolog of *Sl*BBX16, the microProtein 1b (miP1b). A recent study showed that miP1a and miP1b participate in a multiprotein complex which regulates the transcription of the flowering signal *FLOWERING LOCUS T* (*FT*), and flowering time in Arabidopsis (Graeff et al., 2016). To examine whether the Solanaceous‐specific *TCMP‐2* gene could have a general role in reproductive development, we ectopically overexpressed it in Arabidopsis. Arabidopsis plants overexpressing *TCMP‐2* displayed an anticipated flowering phenotype and increased *FT* mRNA level. Similarly, in transgenic tomato plants with increased *TCMP‐2* expression in the flower buds (*pTCMP‐2::TCMP‐1*), we observed an induced expression of the florigen gene *SINGLE FLOWER TRUSS* and an anticipated termination of the sympodial units. Based on these findings, we propose that TCMP‐2 functions in the control of flowering in tomato, most probably by interfering with B‐box microProtein‐containing complexes involved in the regulation of reproductive development.

## 2 MATERIALS AND METHODS

### 2.1 Tomato plant materials and growth conditions

Tomato (*Solanum lycopersicum*) cv UC82 wild‐type plants and two selected independent lines (#1‐2 and #20‐2) transgenic for the *pTCMP‐2::TCMP‐1* genetic construct (Molesini et al., 2018) were used in this study. Seeds were germinated in soil and then seedlings, at the third‐fourth true leaf, after selection with kanamycin (300 mg L^−1^) and confirmation of the transgenic state by PCR, were transplanted in pots (25 cm diameter), and grown in the glasshouse during the springtime. The phenotypic analyses were carried out for two consecutive years.

### 2.2 Yeast two‐hybrid screen

The yeast two‐hybrid (Y2H) screen was performed by Hybrigenics Services, S.A.S., Paris, France (www.hybrigenics‐services.com). A DNA fragment encoding the mature portion of the TCMP‐2 protein (Solyc07g049140; from amino‐acid 53 to 96) was used as bait to screen a tomato (*S. lycopersicum*) fruit cDNA library representative of different fruit developmental stages ranging from mature green to red ripe fruit. 183 positive clones of 30 million tested interactions were selected on a medium supplemented with 10 mM 3‐aminotriazole (3‐AT) to prevent bait autoactivation. A confidence score (PBS, for Predicted Biological Score) was attributed to each interaction following a previously described method (Formstecher et al., 2005). The PBSs were ranked from A (greatest confidence) to D (least confidence). The PBSs positively correlate with the biological significance of interactions (Rain et al., 2001; Wojcik et al., 2002).

To check novel interaction between TCMP‐2 and selected target proteins, the Matchmaker Gold Yeast Two‐Hybrid System (Clontech) was used, following the manufacturer’s instruction with slight modifications. The mature TCMP‐2 protein, representing the bait, was expressed as a fusion to the GAL4 DNA‐binding domain in pGBKT7‐BD vector, and then the recombinant plasmid was introduced into Y2H Gold yeast strain. As prey, full‐length ORFs of *Sl*BBX16, *At*miP1b, *Sl*CO1, *Sl*TPL were cloned in frame into pGADT7‐AD vector and introduced into Y187 yeast strain. Yeast cells were mated, and then the cultures were spread on agar plates followed by incubation at 30°C for 3 days. Growth of yeast on SD/‐Leu/‐Trp medium was used as a control for the presence of both recombinant plasmids, and growth of yeast cells on selection medium (SD/‐Leu/‐Trp/‐His/‐Ade/X‐Gal/Aureobasidin A) plus 10 mM 3‐AT was used to determine positive interactions.

### 2.3 Bimolecular fluorescence complementation assay

The BIFC analysis was performed by Zoonbio Biotechnology Company (www.zoonbio.com). The coding sequence of the mature TCMP‐2 protein and of the entire Solyc12g005750 (*Sl*BBX16) protein was PCR amplified, sequenced, and cloned into the pCAMBIA1300 vector fusing TCMP‐2 to the N‐terminal of YFP and *Sl*BBX16 to the C‐terminal of YFP. The constructs were mobilized into *Agrobacterium tumefaciens* strain GV3101. *A. tumefaciens* cells harboring the recombinant vectors were pelleted and resuspended in infiltration solution (10 mM MgCl_2_, 10 mM MES, pH 5.6, 200 μM acetosyringone) to an OD_600_ value of 0.3‐0.4. Four‐week‐old *Nicotiana tabacum* leaves were co‐infiltrated with the bacteria solutions. 36‐48 hr after agroinfiltration, leaves were visualized using a confocal laser scanning microscope (Zeiss LSM 5Exciter).

### 2.4 *In vivo* Interaction Assay With Ratiometric BIFC (rBiFC)

The coding sequence corresponding to the mature TCMP‐2 was cloned using Gateway system (Thermo Fisher Scientific), into pBiFCt‐2in1_NC or NN allowing for simultaneous cloning of the entire coding sequence of BBX16 or its deletion mutant, indicated as ∆BBX16 (corresponding to the last 49 amino acids), into the same T‐DNA vector backbone and for the ratiometric analysis of the complemented signal due to additional expression of mRFP (Barozzi et al., 2019; Grefen & Blatt, 2012). Constructs were introduced into *A. tumefaciens* cells (strain GV2260), and agroinfiltrations were performed in wild‐type *N. tabacum* as described previously (Paris et al., 2010). Leaves were examined using a confocal laser scanning microscope LSM 710 Zeiss (ZEN Software) mounting material in water (De Caroli et al., 2020). YFP was detected within the short 505–530 nm wavelength range assigning the green color, RFP within 560–615 nm assigning the red color. Excitation wavelengths of 488 and 543 nm were used. The laser power was set to a minimum and appropriate controls were made to ensure that there was no bleed‐through from one channel to the other. Images were processed using Adobe Photoshop 7.0 software (Mountain View).

Fluorescence intensity (complemented YFP vs. RFP) was measured and expressed as ratio index. Complemented signal was weak and dishomogeneous. Seven independent cells were used for quantification of each combination. Images were acquired with similar settings to perform the statistics reported in Figure 2c and the independent samples Student t‐test.

### 2.5 Construction of homology and docking models

The *Sl*BBX16 homology‐based model was built using the Swiss‐Model web server (http://swissmodel.expasy.org). The target sequence was submitted to the server to identify the structural target templates. The first 10 templates with the greatest global quality estimation scores (GMQE; Biasini et al., 2014) were selected for modeling, and the quality of the structures was ranked using the QMEAN scoring function (Benkert et al., 2011). The greatest scoring model, obtained from the structure of the MID1 tandem B‐boxes (PDP code: 2jun), was selected for subsequent docking experiments. The TCMP2‐*Sl*BBX16 complex was generated through protein‐protein docking using the ZDOCK module (http://zdock.umassmed.edu; Pierce et al., 2014). The NMR solution structure of the mature TCMP‐2 tomato peptide (PDB code: 2hlg) was used. The top ten models generated by ZDOCK were compared and the most repetitive structure with the greater ZDOCK score was selected as a representative configuration of the complex.

### 2.6 Northern blot analysis of *SlBBX16*

Total RNA (15 μg) isolated with the NucleoSpin^®^ RNA Plant kit (Macherey‐Nagel) was separated on 1% agarose‐formaldehyde denaturing gel. The gel was blotted overnight on Hybond N^+^ membrane (GE Healthcare) in SSC10X. The DNA probe was labeled with (α‐^32^P)‐dCTP using the "Random primed DNA labeling kit” (Roche). For rapid purification of labeled DNA from unincorporated labeled nucleotides ProbeQuant^™^ G‐50 Micro Columns (GE Healthcare) were used. The membrane was hybridized overnight at 42°C in ULTRAhyb buffer (Ambion). 10^6^ cpm mL^−1^ of labeled probe was added to the hybridization buffer. The membrane was washed two times in 2X SSC/0.1% SDS for 5 min and two times in 0.1X SSC/0.1% SDS for 15 min at 42°C. Autoradiography was then performed using CARESTREAM KODAK BIOMAX XAR film. For *Solyc12g005750* (*SlBBX16*), a DNA probe of 330 nt in length corresponding to the entire protein coding sequence was employed.

### 2.7 Quantitative RT‐PCR analysis

Total RNA was isolated from plant tissues using the NucleoSpin RNA Plant kit (Macherey‐Nagel). cDNA synthesis was performed on 1 µg of total RNA using an oligo(dT) primer and ImProm‐II Reverse Transcriptase (Promega). Quantitative reverse transcription‐PCR was performed using SYBR Green qPCR Supermix‐UDG (Thermo Fisher Scientific) with the StepOnePlus Real‐Time PCR System (Thermo Fisher Scientific). Gene expression levels were normalized to actin expression according to (Livak & Schmittgen, 2001).

### 2.8 Arabidopsis plants expressing *SlTCMP*‐2

For the ectopic overexpression of *SlTCMP‐2* in Arabidopsis, the pCAMBIA1200 vector containing a sequence corresponding to the 96 amino acid‐long coding sequence (from nucleotide 55 to 342 of the TCMP‐2 mRNA NM_001247833) under the control of the CaMV 35S promoter (*35S::TCMP‐2*), was used. The teminator sequence of the gene for the nopaline synthase (NOS) of *A. tumefaciens* was used. The recombinant plasmid was introduced in the *A. tumefaciens* strain GV2260 and transferred into Arabidopsis wild‐type (*Col‐0*) plants through the floral dip method (Zhang et al., 2006). The transgenic plants were identified using hygromycin B (13 μg mL^−1^) in the germination medium (2.15 g L^−1^ MS salts, 0.8% plant agar (w/v), 1% Suc, pH 5.7). The transgenic state was confirmed by PCR using primers spanning the entire genetic cassette, and *TCMP‐2* gene expression was evaluated by reverse transcription‐PCR. For the phenotypic analysis, two selected homozygous *35S::TCMP‐2* transgenic lines (#B2 and #M3) at the T_3_ generation and wild‐type plants were germinated in pots and maintained in a growth chamber at a constant temperature of 25°C under long‐day conditions (16 hr/8 hr light/dark cycle, photosynthetic photon fluence rate of 150 μmol m^−2^ s^−1^ over the waveband 400‐700 nm).

## 3 RESULTS

### 3.1 TCMP‐2 interacts with a member of the B‐box family

**FIGURE 1:**
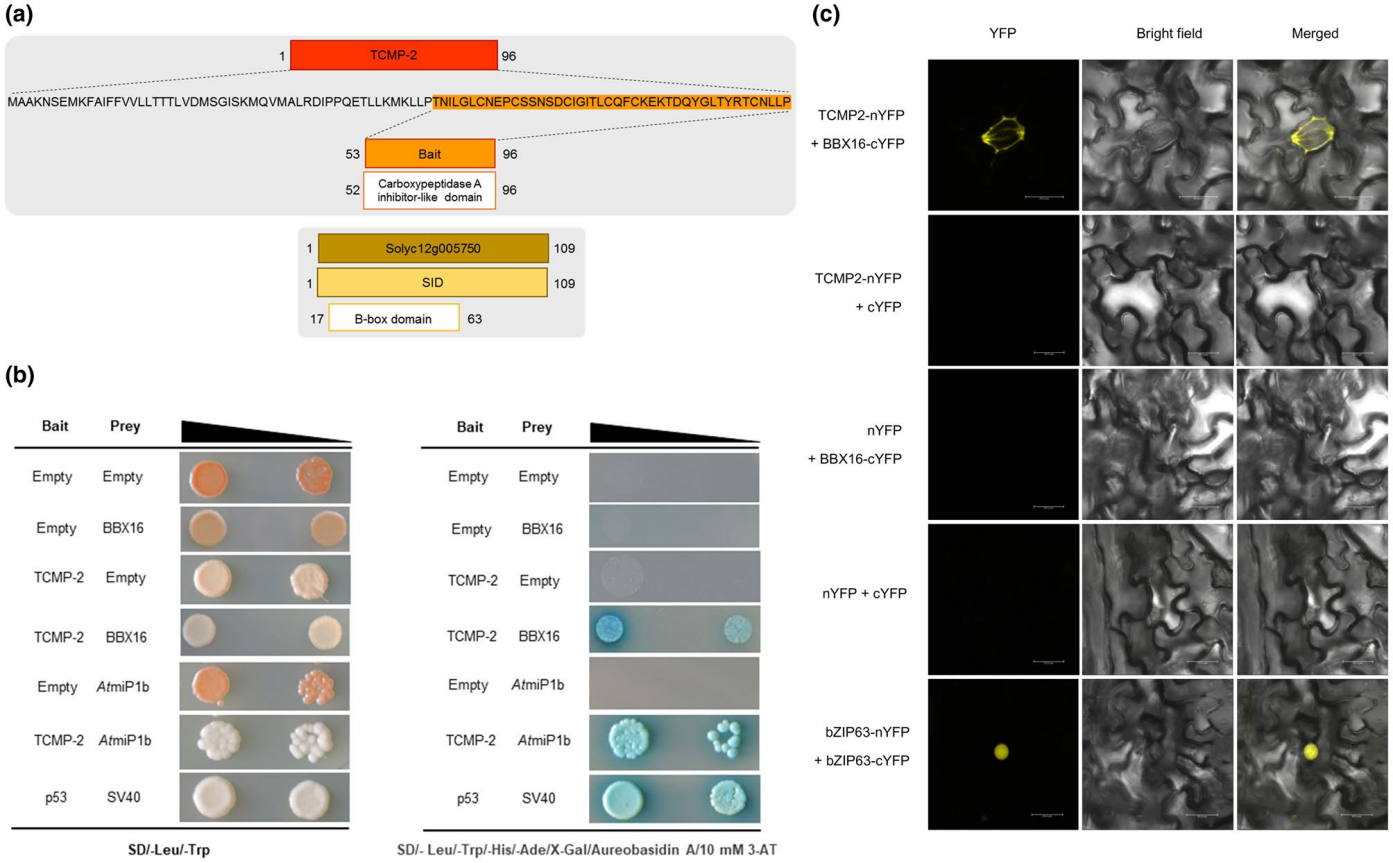
TCMP‐2 interacts with *Sl*BBX16. (a) Schematic representation of the mature TCMP‐2 protein portion used as bait in the Y2H library screen and of its interactor Solyc12g005750, which encodes for a B‐box domain (BBX) containing protein. The selected interaction domain (SID) spans the entire 109 amino acid open reading frame of Solyc12g005750. (b) Y2H analysis of TCMP‐2 interaction with *Sl*BBX16 and *At*miP1b. Yeast cells transformed with different combinations of constructs containing TCMP‐2 fused with the DNA binding domain (BD; bait; TCMP‐2); BBX16 or AtmiP1b fused with the activation domain (AD; prey; BBX16, AtmiP1b) were mated. For negative controls, pGBKT7 without insert (BD alone; Empty) and pGADT7 without insert (AD alone; Empty) were used. For each interaction, two increasing dilutions of the mated cultures were used (10^−2^ and 10^−3^) and spotted on control medium (SD/‐Leu/‐Trp) and on selection medium plates (SD/‐Leu/‐Trp/‐His/‐Ade/X‐Gal/Aureobasidin A/10 mM 3‐AT). Interaction of p53 with SV40 was used as a positive control of the mating system. (c) *Nicotiana tabacum* epidermal cells showing the interaction between TCMP‐2 and BBX16. *A. tumefaciens* cells harboring the mature TCMP‐2 fused with the N‐terminus of YFP (nYFP) and the *Sl*BBX16 fused with the C‐terminus of YFP (cYFP) were co‐infiltrated into tobacco leaves. nYFP and cYFP empty vectors were used as negative controls; the combination of bZIP63‐nYFP and bZIP63‐cYFP was used as a positive control. The cells were imaged by confocal microscopy 36‐48 hr later. Scale bars indicate 25 µm

**FIGURE 2:**
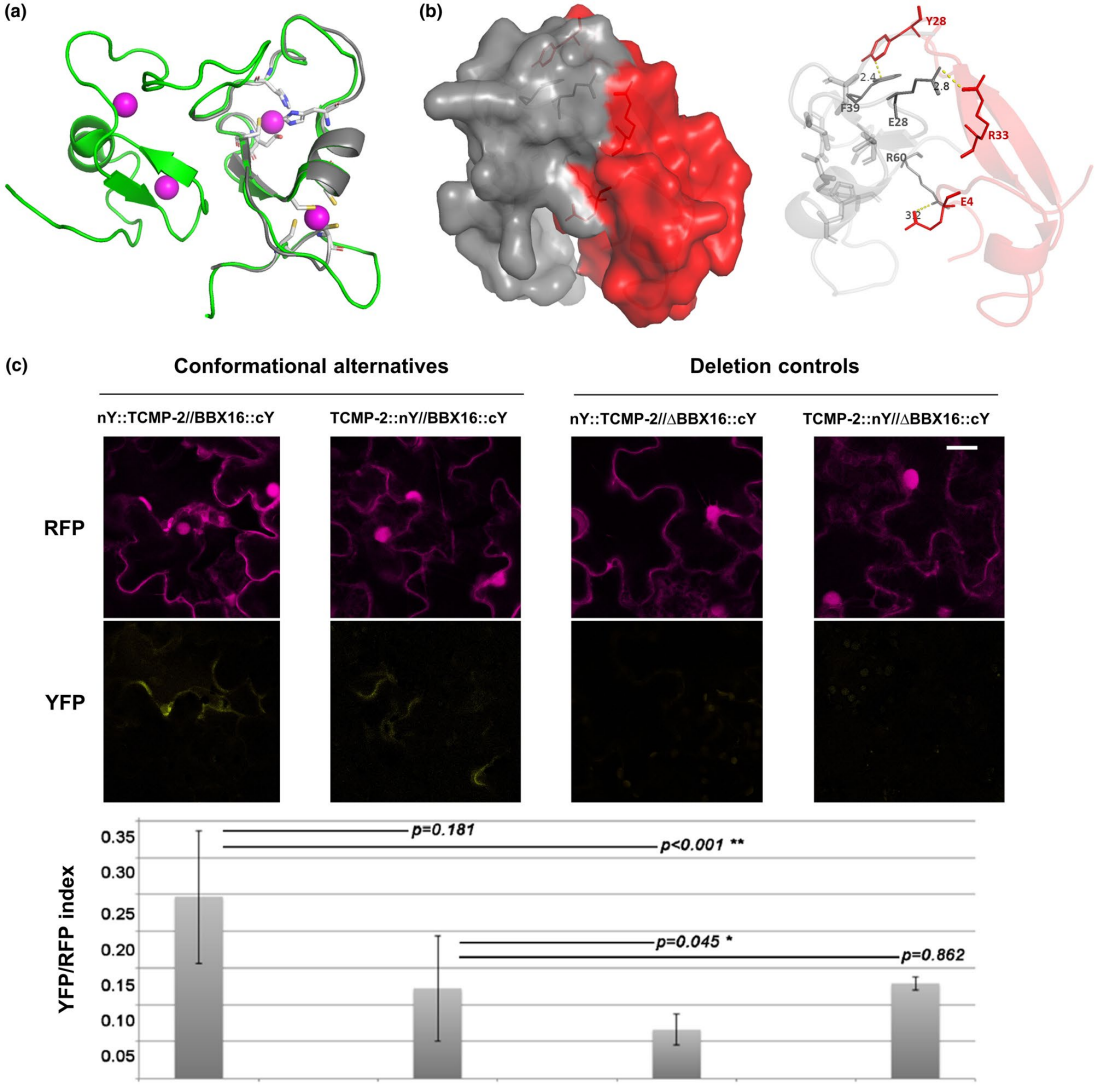
*Sl*BBX16 homology and docking models. (a) *Sl*BBX16 homology model. Superimposed three‐dimensional model of *Sl*BBX16 (in grey) and the NMR structure template of the Midline‐1 tandem B‐boxes (2 jun) shown in green. The Zn ions found in the Midline‐1 NMR structure are shown as purple spheres. The conserved amino acid residues coordinating the Zn ions in *Sl*BBX16 are shown as sticks. (b) Molecular docking complex employing ZDOCK program. Three‐dimensional structure of the *Sl*BBX16‐TCMP‐2 complex with solid surfaces representing the interface (left), and main residues involved in the protein‐protein interaction (right). *Sl*BBX16 and TCMP‐2 are shown in grey and red respectively. Residues involved in the binding interface are shown as sticks, and the interatomic distances are indicated as yellow dotted lines and in Angstrom respectively. (c) Quantitative rBIFC analysis of TCMP‐2 interaction with BBX16 with different control deletion constructs. The higher row of images shows representative tobacco epidermal cells transiently transformed and expressing the cytosolic reference RFP; below the YFP complemented signal, corresponding to the indicated construct combination is shown; scale bar = 20 μm. YFP/RFP fluorescence intensities from 7 different independent samples were calculated as the average YFP/RFP ratio. Independent samples Student’s t‐test was applied (*p < 0.05)

TCMP‐2 is a member of a small family of tomato cystine‐knot proteins whose expression is modulated during flower and fruit development. To clarify TCMP‐2 mode of action, a high‐throughput yeast two‐hybrid (Y2H) screen was conducted to identify interacting partners . Among the tomato cDNA libraries available, we chose a fruit cDNA library since TCMP‐2 is greatly expressed in the later stages of flower/fruit development (Molesini et al., 2018). Using the mature TCMP‐2 protein as bait, we were able to identify 47 potential interacting partners which were categorized into C and D classes (Table S1). One of these was a “C” ranked interactor (Solyc12g005750) of 109 amino acids, annotated as a B‐box Zinc finger CONSTANS‐LIKE 4 protein (Figure 1a), and closely related to the Arabidopsis microProtein 1b (*At*miP1b). Hereafter, the protein product encoded by Solyc12g005750 is indicated as *Sl*BBX16 following the classification of Chu and collaborators (Chu et al., 2016). In the Y2H screen, this protein was isolated five times with two independent clones. The selected interaction domain (SID) of the Solyc12g005750 protein, responsible for the interaction with TCMP‐2, covers the entire amino acid sequence (Figure 1a). We demonstrated by Y2H that TCMP‐2 is able to interact with both *Sl*BBX16 and *At*miP1b (Figure 1b). For this purpose, the nucleotide sequence containing the cystine‐knot motif and corresponding to the 44 amino acid‐long TCMP‐2 mature peptide, representing the bait, was expressed as a fusion to the GAL4 DNA‐binding domain in the Y2HGold strain. As prey, we used the full‐length ORFs of either *Sl*BBX16 or *At*miP1b, which was fused in frame to the Gal4‐activation domain, and the resulting recombinant vector was used to transform the Y187 strain (Figure 1b). To test the interaction between TCMP‐2 and *Sl*BBX16 *in planta*, we performed a bimolecular fluorescence complementation (BiFC) analysis (Walter et al., 2004) via transient expression in *Nicotiana tabacum* epidermal cells. Confocal laser scanning microscopy images showed the reconstitution of the yellow fluorescent protein (YFP) confirming that the TCMP‐2 protein can physically interact with *Sl*BBX16 *in vivo* (Figure 1c). To support the experimental data, we exploited computational modeling as a tool to define the interaction between TCMP‐2 and *Sl*BBX16. The three‐dimensional structural model of *Sl*BBX16 was obtained by a homology modeling strategy using the Midline‐1 structure (PDB code: 2jun), which contains two B‐box domains. As shown in Figure 2a, the tertiary structural alignment of *Sl*BBX16 with the template reveals that the model aligned structurally with the B‐box2 domain of Midline‐1. The structural model contains the consensus residues of the two metal‐binding sites, having four Cys residues that would coordinate one zinc (Zn) ion, and a Cys‐Asp‐His‐His motif in the second Zn‐binding site. The stereochemical quality analysis of residues in the model was carried out using Ramachandran Plot. In the generated model, the percentage of residues in the most favored regions and additional allowed regions was 88.1% and 9.5%, respectively, and only 2.4% of the residues were in the disallowed region, indicating the good quality of the model. To provide the ability of the B‐box domain in *Sl*BBX16 to interact with TCMP‐2, we used the rigid‐body protein‐protein docking program ZDOCK. The binding interface and the key residues involved in the formation of the *Sl*BBX16‐TCMP‐2 complex are shown in Figure 2b. Two acidic and a hydrophobic residue of the proteins appear involved at the interface (Figure 2b, right panel). The oxygen atoms of the Glu28 and Arg60 side chains of *Sl*BBX16 likely establish a salt bridge with side‐chain nitrogen atoms of Arg33 and Glu4 of TCMP‐2, respectively. Phe39 could provide additional stabilizing energy through π‐stacking interactions with the Tyr28 of TCMP‐2. This result indicated that the B‐box domain alone of *Sl*BBX16 is likely responsible for the interaction with the TCMP‐2. We performed a ratiometric BIFC (rBIFC), using as negative control of the interaction a deleted version of the *Sl*BBX16 protein (hereafter indicated as ΔBBX16), lacking the first sixty amino acids (the protein region containing the characteristic residues of the B‐box domain). Four different combinations of constructs for rBIFC were performed, considering two conformational alternatives differing in the position of TCMP‐2 (cloned either downstream or upstream of the N‐terminus of YFP, i.e. nYFP::TCMP‐2//BBX16::cYFP and TCMP‐2::nYFP//BBX16::cYFP). BBX16 is fused upstream to the C‐terminus of YFP in both constructs (Figure 2c). The controls (i.e. nYFP::TCMP‐2//ΔBBX16::cYFP and TCMP‐2::nYFP//ΔBBX16::cYFP) contain the mutated *Sl*BBX16 (Figure 2c). The interactions appeared generally weak, indicating some uncharacterized limitations to the efficient interaction of protein partners but a significantly stronger signal was revealed for the combination nYFP::TCMP‐2//BBX16::cYFP (Figure 2c). The signal for this combination was highly variable in different cells so that, even if higher, was not significantly different from the signal of the conformational alternative TCMP‐2::nYFP//BBX16::cYFP. The differences with deletion controls were statistically significant, indicating that the removal of the B‐box domain of *Sl*BBX16 (Figure 2a and b) restrains the interaction. Only in the case of the combination nYFP::TCMP‐2//BBX16::cYFP, it was possible to observe fluorescent signals compatible with cellular membranes (Figure 2c and Figure S1), but signal weakness suggests that further investigations are required to define the localization of the interacting proteins.

### 3.2 *Sl*BBX16 is a homolog of the flowering‐related Arabidopsis miP1b

**FIGURE 3:**
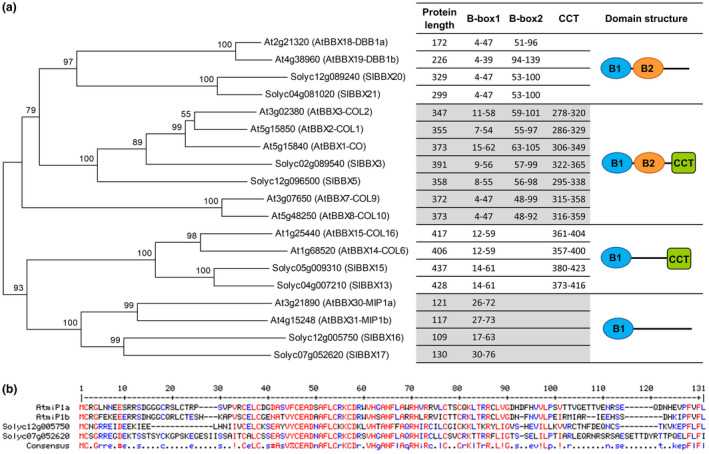
Sequence comparison of tomato and Arabidopsis BBX proteins. (a) Phylogenetic tree of tomato and Arabidopsis BBX family members and structural domains of the BBX proteins. A total of nineteen protein sequences were aligned in MEGA 5 (Tamura et al., 2011), using the default ClustalW algorithm. The evolutionary history was inferred using the Minimum Evolution method (Rzhetsky & Nei, 1992). The optimal tree with the sum of branch length = 307.80664062 is shown. The percentage of replicate trees in which the associated taxa clustered together in the bootstrap test (500 replicates) are shown above the branches (Felsenstein, 1985). The tree is drawn to scale, with branch lengths in the same units as those of the evolutionary distances used to infer the phylogenetic tree. The evolutionary distances were computed using the number of differences method (Nei & Kumar, 2000) and are in the units of the number of amino acid differences per sequence. The ME tree was searched using the Close‐Neighbor‐Interchange (CNI) algorithm (Nei & Kumar, 2000) at a search level of 1. The Neighbor‐joining algorithm (Saitou & Nei, 1987) was used to generate the initial tree. The analysis involved 19 amino acid sequences. All positions containing gaps and missing data were eliminated. There was a total of 86 positions in the final dataset. For each BBX protein in the phylogenetic tree, the corresponding amino acid length, the position of the conserved domain/s identified using the SMART (Simple Modular Architecture Research Tool) web resource (http://smart.embl.de; Letunic & Bork, 2018), and the schematic drawing of the domain structure are indicated. The *Sl*BBX16, *Sl*BBX17, *At*miP1a, and *At*miP1b proteins characterized by the presence of a single B‐box domain cluster together. (b) Alignment created by Multalign (Corpet, 1988) of the *Sl*BBX16 (Solyc12g005750), *Sl*BBX17 (Solyc07g052620), *At*miP1a (*At*BBX30), and *At*miP1b (*At*BBX31) proteins. Conserved residues are coloured in red (high consensus level 90%) and in blue (low consensus level 50%). A position with no conserved residues is represented by a dot in the consensus line. The consensus symbols are ! (IV), % (FY), and # (NDE)

*Sl*BBX16 belongs to the B‐box (BBX) Zn finger protein family characterized by the presence of one or more B‐box domains, whose tertiary structure is stabilized by Zn ion binding. The tomato and Arabidopsis BBX families can be divided into subfamilies according to their protein structures (Chu et al., 2016; Khanna et al., 2009). Phylogenetic comparison carried out with tomato and Arabidopsis members of the different subfamilies (Figure 3a and Table S2), revealed that the closest *Sl*BBX16 homolog in Arabidopsis is miP1b (named also *At*BBX31; Graeff et al., 2016), which shows a 48% amino acid sequence identity (Figure 3b). *Sl*BBX16 is also very similar (45% identical) to a second BBX protein of Arabidopsis, the *At*miP1a (referred to as *At*BBX30). Among the BBX tomato family, the closest homolog (i.e. 46% identity) of *Sl*BBX16 is *Sl*BBX17 (Solyc07g052620; Figure 3b).

*Sl*BBX16, *Sl*BBX17, *At*miP1a, and *At*miP1b belong to the same group characterized by the presence of only one B‐box domain (CX_2_CX_8_CX_7_CX_2_CX_4_HX_8_H) predicted to mediate protein‐protein interactions (Gangappa & Botto, 2014; Graeff et al., 2016; Khanna et al., 2009) and by the lack of a CCT domain, which is associated with a role in transcriptional regulation and nuclear transport (Gendron et al., 2012; Yan et al., 2011; Figure 3a). Because of the absence of the CCT domain, the principal function of these proteins is to interfere with the formation of protein complexes (Gangappa & Botto, 2014; Graeff et al., 2016). Recently, the *At*miP1a/b were proved to engage CONSTANS (CO), a positive regulator of flowering time (Graeff et al., 2016), in a repressor complex with TOPLESS (TPL) determining the inhibition of *FLOWERING LOCUS T* (*FT*) expression (Graeff et al., 2016). The overexpression of either *At*miP1a or *At*miP1b resulted in delayed flowering by inhibiting the transcription of the florigen gene *FT* under long‐day conditions (Tiwari et al., 2010; Valverde et al., 2004).

### 3.3 The ectopic overexpression of *TCMP‐2* in Arabidopsis affects the flowering time

**FIGURE 4:**
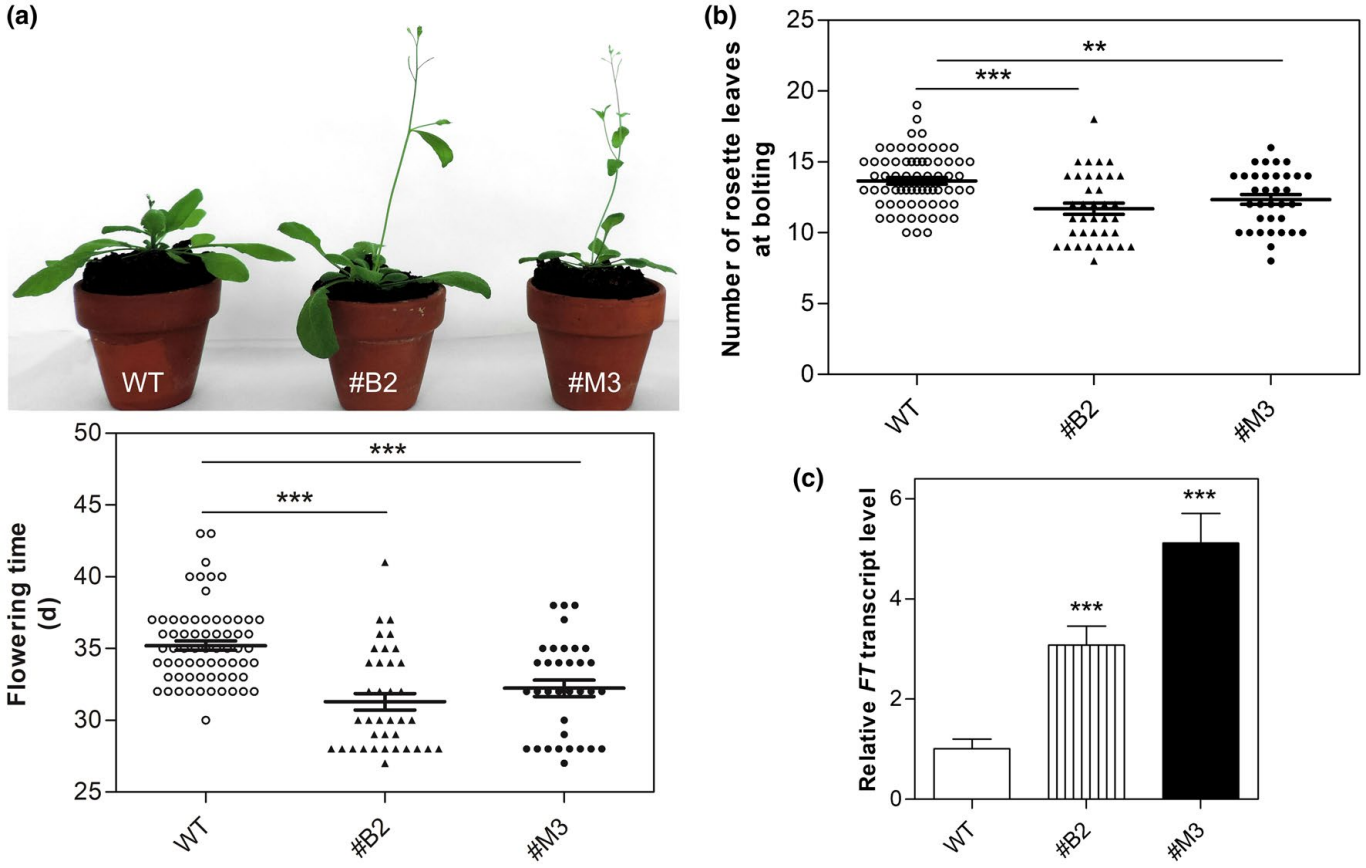
Arabidopsis plants overexpressing *TCMP‐2* display early flowering time. (a) Picture representative of the early flowering phenotype displayed by the *35S::TCMP‐2* lines (#B2 and #M3) compared to a wild‐type plant at the same age (upper panel). Quantification of the flowering time by counting the day from germination at which each plant reaches bolting (lower panel). (b) Number of rosette leaves produced at bolting. The values reported in panels (a) and (b) are means ± SE (n ≥ 34 plants). (c) Expression level of *FT* in leaves of wild‐type and #B2 and #M3 transgenic lines. The values reported are means ± SE (n = 3). Student’s t‐test was used to compare differences between transgenic and wild‐type plants (**p < 0.01; ***p < 0.001)

As described above, Y2H demonstrated that TCMP‐2 can interact with *At*miP1b (Figure 1b). Then, we decided to ectopically overexpress the Solanaceous‐specific *TCMP‐2* gene in Arabidopsis to determine whether it can interfere with endogenous pathways controlling reproductive development. We phenotypically characterized two selected independent *35S::TCMP‐2* lines (#B2 and #M3; Figure S2) and found that in both lines bolting occurred on the average 3‐4 d earlier as compared to wild‐type plants (Figure 4a). The number of rosette leaves at bolting was reduced in both transgenic lines (Figure 4b). Since the transition from vegetative to reproductive growth is controlled in Arabidopsis by multiple molecular pathways, which converge on the induction of florigen gene, we analysed the expression of *FT* (*At1g65480*) in rosette leaves of wild‐type and *35S::TCMP‐2* plants. The transcript level of *FT* was approximately 3‐ and 5‐fold increased in #B2 and #M3 transgenic lines, respectively, as compared with the wild‐type (Figure 4c). The early flowering phenotype observed in Arabidopsis plants overexpressing TCMP‐2 resembles that seen when *At*mip1a/b were silenced (Graeff et al., 2016).

### 3.4 *Sl*BBX16 expression in tomato plants

**FIGURE 5:**
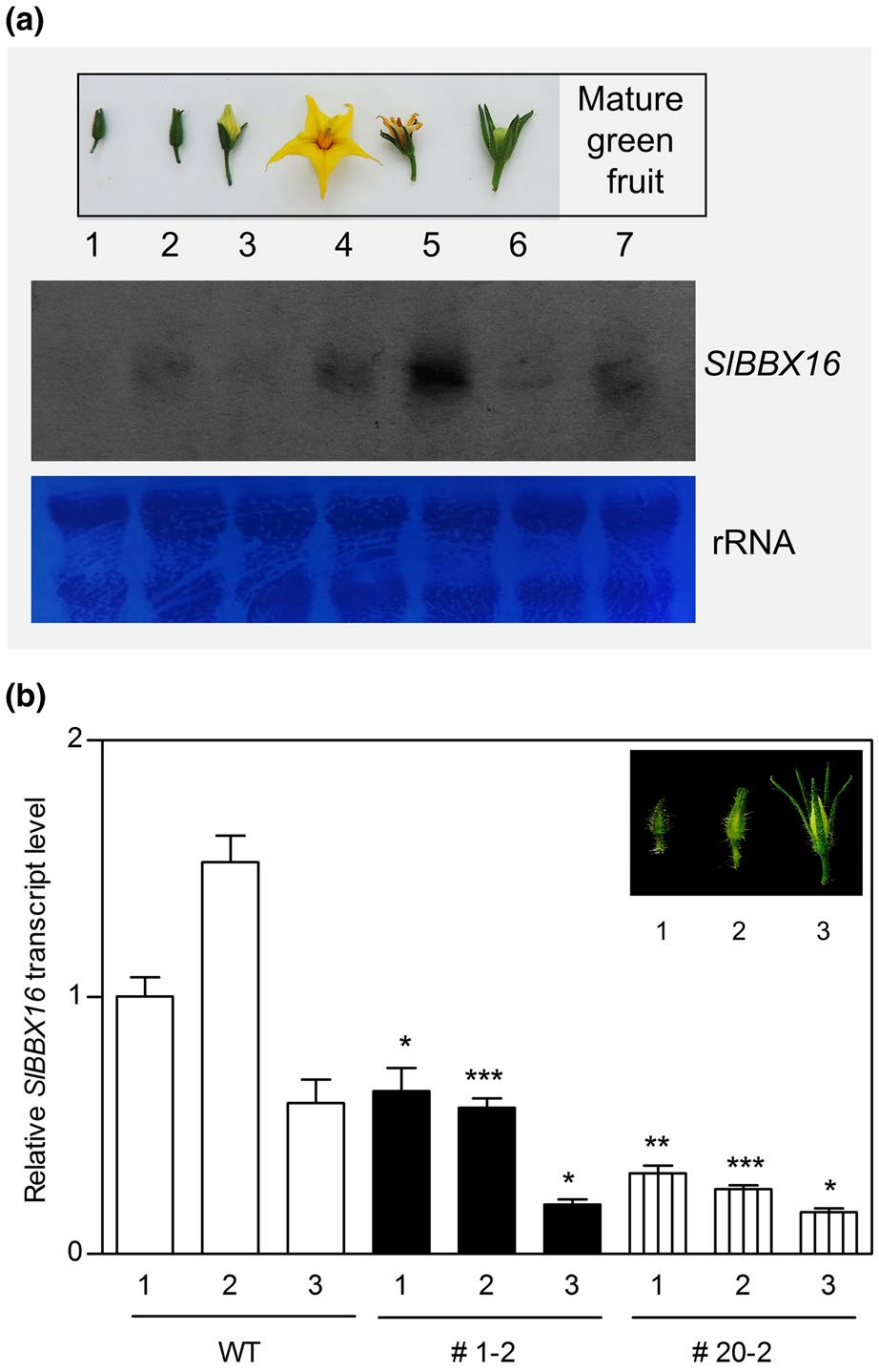
*SlBBX16* expression in wild‐type and *pTCMP‐2::TCMP‐1* transgenic plants. (a) Northern blot analysis of *SlBBX16* mRNA at different stages of flower/fruit development. 1, flower bud 0.5‐0.6 cm long; 2, flower bud 0.7‐0.9 cm long; 3, flower bud 1.0‐1.1 cm long (1‐3 days before anthesis); 4, anthesis/1 day post anthesis; 5, 4‐5 days post anthesis; 6, ovary 0.5‐1 cm long; 7, mature green fruit. Staining with 0.1% toluidine blue of the rRNA is shown as control for homogenous transfer of the loaded RNA samples. (b) *SlBBX16* expression level in flower buds of stages 1‐3 collected from wild‐type and *pTCMP‐2::TCMP‐1* transgenic lines (#1‐2 and #20‐2). The values are means ± SE (n = 3). Student’s t‐test was used to compare differences between transgenic and wild‐type flower buds at the same developmental stage (*p < 0.05; **p < 0.01; ***p < 0.001)

To confirm the likelihood of the TCMP‐2/*Sl*BBX16 interaction during flower bud development, we examined the expression pattern of *SlBBX16* in seven representative reproductive developmental phases, including flower buds before anthesis (stages 1‐3), flowers at anthesis (stage 4), fertilized flowers (stage 5), ovaries (0.5‐1 cm long; stage 6), and mature green fruits (stage 7; Figure 5a). *SlBBX16* was expressed in all the developmental stages analysed, exhibiting a fluctuating expression (Figure 5a). The analysis of the *TCMP‐2* expression in the same developmental stages reported in previous works (Cavallini et al., 2011; Molesini et al., 2018) indicates that the two genes can interact both during flower buds development as well as in young fruits. To verify whether increased level of TCMP‐2 during early flower development (stages 1‐3) could result in *SlBBX16* perturbation, we monitored the expression of *SlBBX16* in *pTCMP‐2::TCMP‐1* transgenic plants (#1‐2 and #20‐2 lines; Molesini et al., 2018). We found that *SlBBX16* expression in preanthesis flower buds was differently modulated as compared with wild‐type plants, with a decline in the expression ranging from 40 to 80% in flower buds of both #1‐2 and #20‐2 transgenic lines (Figure 5b).

### 3.5 Tomato plants with increased *TCMP‐2* level in flower buds displayed an anticipated termination of the sympodial units

We previously generated *pTCMP‐2::TCMP‐1* transgenic plants altering the relative expression of *TCMP‐1* and *TCMP‐2* with the aim of monitoring the effects on fruit development. These plants showed an anticipated production of fruits (Molesini et al., 2018). An unexpected molecular effect of this genetic manipulation was the marked increase of *TCMP‐2* mRNA level in the flower buds before anthesis. Comparing with wild‐type, *TCMP‐2* expression reached in the two transgenic lines (#1‐2 and #20‐2) a 100‐ and 30‐fold increase respectively (Molesini et al., 2018). Considering that TCMP‐2 interacts with a BBX protein known to regulate flowering time in Arabidopsis and its overexpression in this species caused an anticipated flowering and increase in *FT* expression, we thought that *pTCMP‐2::TCMP‐1* plants could be altered in the flowering pattern. Therefore, we sought to reconsider the phenotype of these transgenic plants focusing on the transition from vegetative to reproductive phase and assay the expression of genes regulating flowering pattern in tomato. Differently from Arabidopsis, which is characterized by monopodial shoot architecture, in which the apical meristem, being indeterminate, is active throughout the entire plant life cycle, in tomato, the transition to the reproductive stage is more complex and follows a sympodial growth. The growth of the primary shoot is terminated with the first inflorescence, and shoot growth continues from specialized axillary meristems, called sympodial meristems. Reiterated sympodial units (SUs) are then formed from sympodial meristems on the main and lateral axis (Park et al., 2012; Soyk et al., 2017).

**FIGURE 6:**
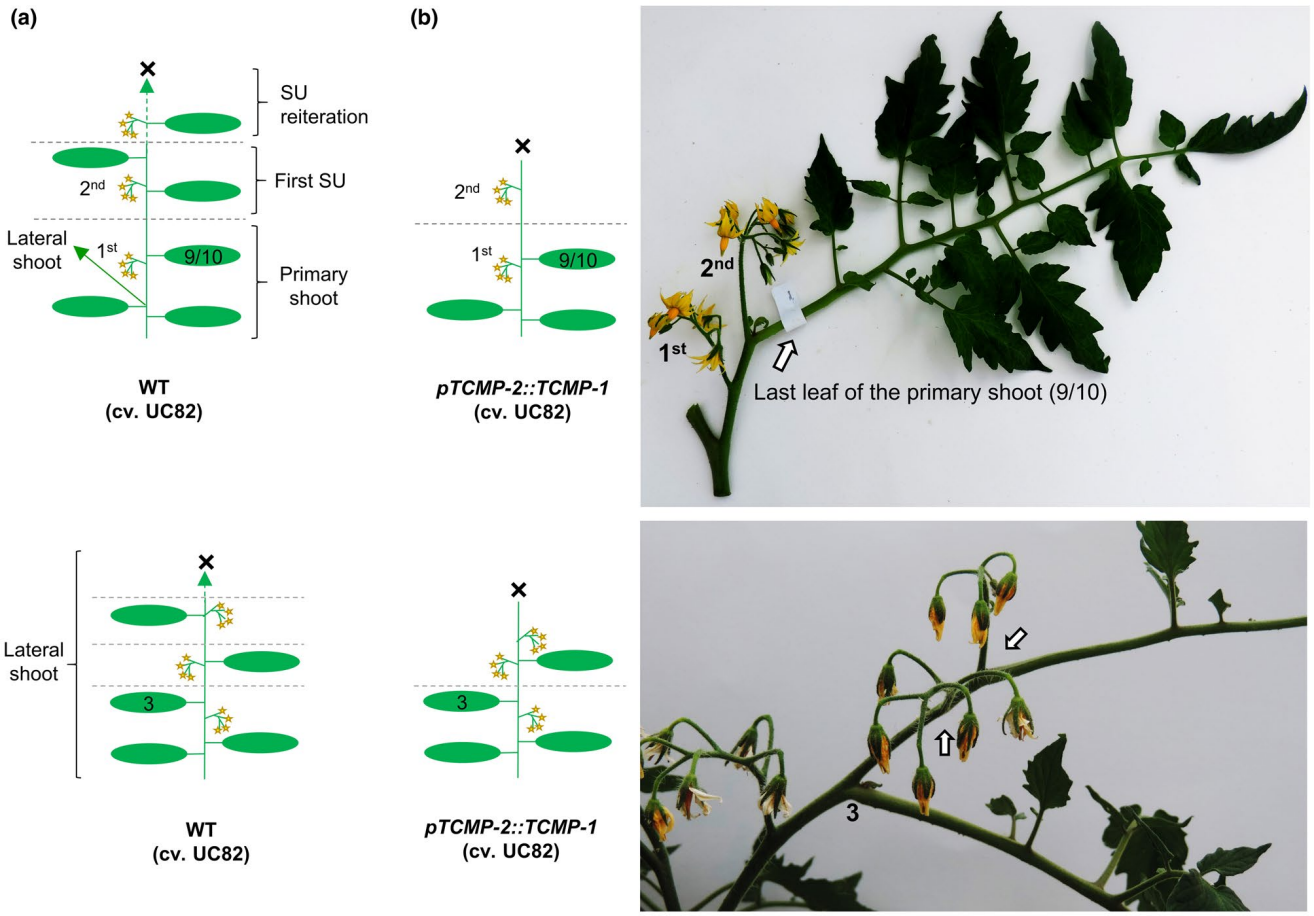
Shoot architecture of *pTCMP‐2::TCMP‐1* plants. (a) Schematic diagram showing shoot architecture of a wild‐type (WT) determinate tomato plant cv UC82. (Upper panel), in WT plants, the primary shoot meristem is terminated by the first inflorescence after 9‐10 leaves. Reiterated sympodial units (SUs) are then formed from sympodial meristems. The sympodial cycling accelerates progressively causing leaf production to decrease in successive units until growth ends (marked with “X”). The green arrow indicates a lateral shoot. (Lower panel), in WT plants, lateral shoot displays a first SU which consists of three leaves and an inflorescence, and subsequent SUs form fewer leaves until the shoot is terminated. (b) Schematic diagram showing shoot architecture displayed by the transgenic *pTCMP‐2::TCMP‐1* plants and relative pictures showing alterations in the flowering pattern

The transgenic *pTCMP‐2::TCMP‐1* tomato plants were obtained by genetic transformation of the determinate UC82 cultivar (Jones et al., 2007), whose inflorescence architecture is schematically depicted in Figure 6. Wild‐type UC82 plants terminate with a primary inflorescence after about 9‐10 leaves. The first SU consists of two leaves and an inflorescence (second inflorescence). The sympodial cycling accelerates progressively causing leaf production to decrease in successive units until growth ends (Figure 6a, upper panel). The lateral shoot which originates from axillary buds displays a first SU which consists of three leaves and a terminal inflorescence, and subsequent SUs form fewer leaves until the shoot is terminated (Figure 6a, lower panel). In *pTCMP‐2::TCMP‐1* plants, the first inflorescence is formed after the same number of leaves as in wild‐type (Figure 6b). However, in approximately 70% of transgenic plants, we observed changes in flowering pattern. Concerning the main shoot, some of the transgenic plants did not show activation of the sympodial cycle (Figure 6b, upper panel). After the primary inflorescence, the shoot terminates with a second inflorescence without sympodial cycle activation. In other transgenic plants, irregular SUs were observed (Figure S3). Concerning the architecture of lateral shoots, irregular formation of the SUs was observed in some plants of both transgenic lines, with a tendency of reduction in the number of leaves between inflorescences compared to wild‐type (Figure 6b, lower panel). Collectively, the alterations observed in transgenic plants were related to an anticipated termination of the SU.

**FIGURE 7:**
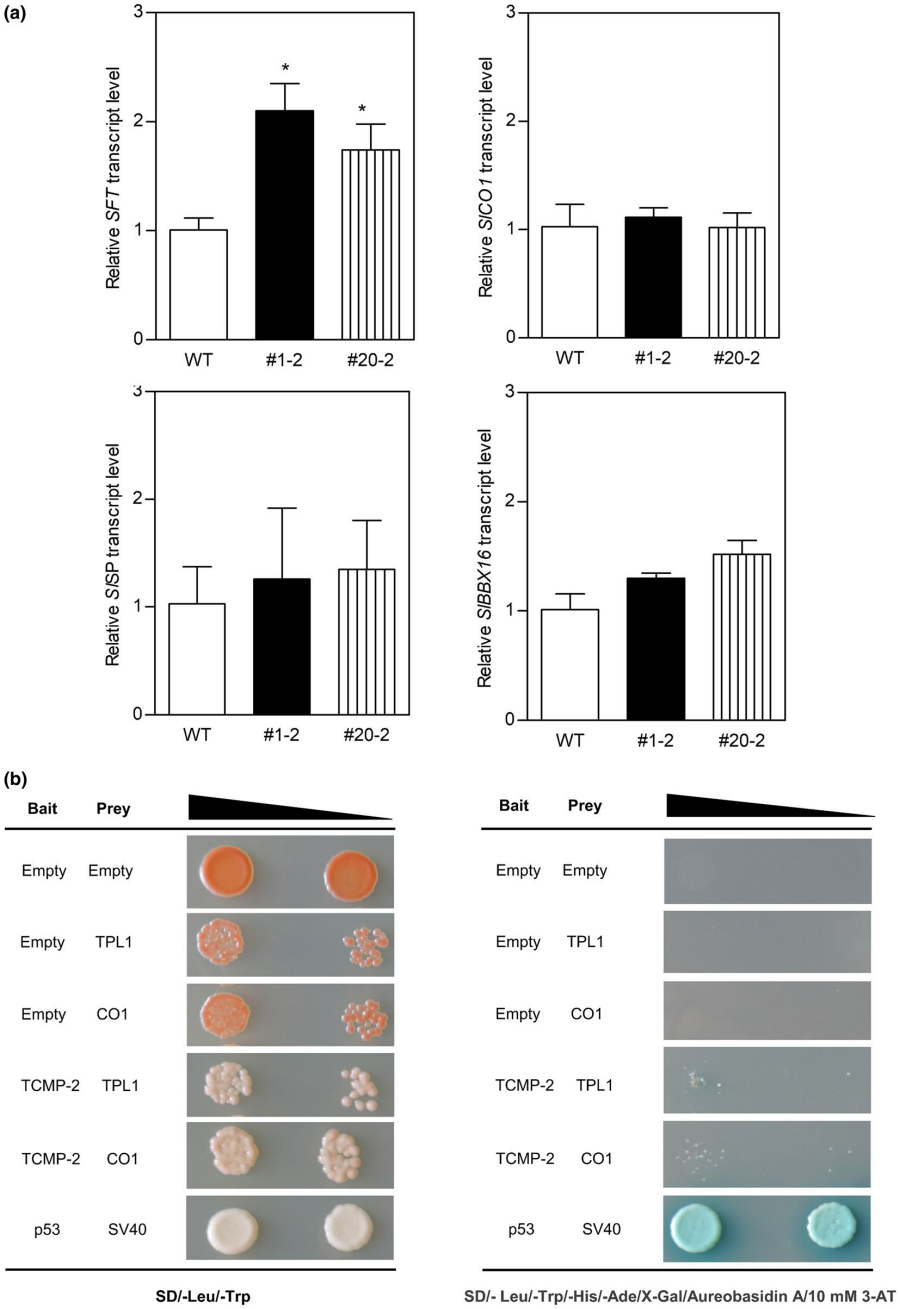
Molecular changes associated with anticipated sympodial termination in *pTCMP‐2::TCMP‐1* plants. (a) Expression levels of *SFT*, *SlCO1*, *SlSP*, and *SlBBX16*, in wild‐type and #1‐2 and #20‐2 lines. The values reported are means ± SE (n = 3). Each biological sample was obtained from RNA of 3 pools of either leaf (*SFT*, *SlCO1*, and SlBBX16) or meristem (*SlSP*) tissues collected from seven plants. Student’s t‐test was used to compare the differences in transcript level in transgenic plants versus wild‐type (*p < 0.05). (b) Y2H interaction assay between TCMP‐2 and *Sl*CO1 and *Sl*TPL1. For negative controls, pGBKT7 without insert (BD alone; Empty), pGADT7 without insert (AD alone; Empty) were used. For each protein‐protein interaction, two increasing dilutions of the mated cultures were used (10^−2^ and 10^−3^) and spotted on control medium (SD/‐Leu/‐Trp) and on selection medium plates (SD/‐ Leu/‐Trp/‐His/‐Ade/X‐Gal/Aureobasidin A/10 mM 3‐AT). Interaction of p53 with SV40 was used as a positive control of the mating system

Sympodial cycling is controlled by a balance between the activity of two antagonistic genes: flower‐promoting (SINGLE FLOWER TRUSS; SFT) and flower‐repressing (SELF‐PRUNING; *Sl*SP) (Lifschitz et al., 2014). Considering the altered SU pattern observed in *pTCMP‐2::TCMP‐1* plants, we analysed in leaves of transgenic and wild‐type plants the expression of the florigen *SFT* (*Solyc03g063100*), and the closest homolog of the Arabidopsis *CONSTANS* (*SlCO1; Solyc02g089540*), and in the meristems the expression of the antiflorigen *SlSP* (*Solyc06g074350*; Figure 7a). The transcript levels of *SlSP*, and *SlCO1* did not show variations, whereas *SFT* expression was on the average doubled in both transgenic lines as compared with wild‐type (Figure 7a). In the leaves of *pTCMP‐2::TCMP‐1* plants, we did not observe changes in *SlBBX16* expression (Figure 7a). *TCMP‐2* mRNA level was barely detectable by qRT‐PCR analysis in the aerial vegetative tissues of *pTCMP‐2::TCMP‐1* transgenic plants (data not shown).

In Arabidopsis, *At*miP1b interacts with CO mediating its recruitment into a TPL trimeric complex, thus hampering the activation of *FT* by CO and leading to flowering repression (Graeff et al., 2016). In tomato, the functional characterization of *Sl*CO1 revealed no obvious flowering time phenotype (Ben‐Naim et al., 2006) and a role for *Sl*TPL1 (Solyc03g117360), the closest homolog of the Arabidopsis TPL gene, in reproductive development has not yet been described in tomato. To gain more information on the interacting network of TCMP‐2, we investigated by Y2H whether TCMP‐2 interacts with *Sl*CO1 and *Sl*TPL1 proteins. Our data indicate that TCMP‐2 cannot directly bind either to CO1 or TPL1 (Figure 7b).

## 4 DISCUSSION

In flowering plants, the transition from vegetative to reproductive growth is a tightly regulated process which depends on environmental cues, such as day length and temperature, as well as on endogenous signals (Blümel et al., 2015; Lifschitz et al., 2014). Among the endogenous signals, besides the role played by classical phytohormones as mediators of cell‐to‐cell interactions, a growing body of evidence reveals a role for small secreted peptides in signaling over short and long distances (De Smet et al., 2009; Marshall et al., 2011). Secreted signaling peptides can be divided into two major categories: small post‐translationally modified peptides and Cys‐rich peptides (CRPs), containing typically 6‐8 Cys residues forming intramolecular disulfide bonds (Matsubayashi & Sakagami, 2006). Plant peptides act at different levels during reproductive development; for instance, some CRPs expressed specifically in flowers, control the communication between male and female reproductive organs and gametic cells during fertilization (Dresselhaus & Franklin‐Tong, 2013; Ingram & Gutierrez‐Marcos, 2015; Joly et al., 2019; Pease et al., 2016; Qu et al., 2015). Recently, some CRP peptides/miniproteins, i.e. the tomato TCMP‐1 and TCMP‐2, were also implicated in fruit formation (Molesini et al., 2018). TCMPs are classified as cystine‐knot miniproteins due to the peculiar Cys bond arrangement, and belong to a subgroup found only in species of the *Solanaceae* family (Molesini et al., 2017; Postic et al., 2018). The endogenous levels and ratio of TCMP‐1 and TCMP‐2 in flower buds and fertilized ovaries affect fruit development, as increased *TCMP‐2* expression before anthesis results in early fruit production (Molesini et al., 2018). The *TCMP‐1* and *TCMP‐2* genes are regulated by a common YABBY‐type transcription factor, named INNER NO OUTER (Molesini et al., 2018), for which a role in ovule development has been demonstrated (Skinner et al., 2016; Villanueva et al., 1999). This common co‐regulation might contribute to the maintenance of relative levels of both proteins during the transition from flowers to fruits.

The in‐depth phenotypic analysis of *pTCMP‐2::TCMP‐1* plants has now revealed that the increased expression of TCMP‐2 in pre‐anthesis flower buds alters the flowering pattern, besides the fruit initiation capacity, resulting in an accelerated shoot growth termination. As known, the transition to flowering is synonymous with termination in tomato (Lifschitz et al., 2014). Tomato produces flowers irrespective of the photoperiodic conditions (i.e. day‐neutral), although other environmental factors such as light intensity and ambient temperature can affect flowering (Calvert, 1964). Flowering is induced in tomato, as in the photoperiod‐sensitive plants, by a mobile florigen signal called SINGLE FLOWER TRUSS (SFT), while sympodial cycling in tomato is regulated by the ratio between SFT (florigen) and SELF‐PRUNING (SP; antiflorigen; Lifschitz et al., 2014). In accordance with the anticipated termination phenotype, in *pTCMP‐2::TCMP‐1* plants *SFT* expression in the leaves is induced, whereas *SP* expression in the meristems is unaltered. The increased expression of *SFT* in the leaves, which normally correlates with an increase in the corresponding protein product, results in a higher amount of the protein transported to the meristem where SFT antagonizes the *Sl*SP action. On the other hand, since *SlSP* mRNA level in the meristem did not change in *pTCMP‐2::TCMP‐1* plants, it is likely that SFT/SP protein ratio increases. It has been proposed that shifts to shoot termination in tomato are mediated by dynamic SFT/SP ratio that depends on the imported florigen (*SFT*) in the meristem (Lifschitz et al., 2014).

Interestingly, TCMP‐2 interacts *in vitro* and *in vivo* with *Sl*BBX16, a member of the B‐box (BBX) protein family. This group of proteins is known to participate in photomorphogenesis, UV‐B protection, and photoperiodic flowering control (Graeff et al., 2016; Heng et al., 2019; Song et al., 2020; Yadav et al., 2019). The closest *Sl*BBX16 homolog in Arabidopsis is the microProtein 1b (miP1b), also referred to as *At*BBX31 (Graeff et al., 2016). Both *Sl*BBX16 and *At*miP1a/b present a small size (i.e. <140 amino acids), a single BBX domain, and lack the CCT domain typical of BBX transcription factors suggesting that they act as microProteins, which regulate multiprotein complexes at the post‐translational level (Bhati et al., 2018; Eguen et al., 2015; Staudt & Wenkel, 2011). The overexpression of *At*miP1a (*AtBBX30*) and *AtBBX31* in Arabidopsis resulted in flowering repression by inhibiting the transcription of the florigen gene *FLOWERING LOCUS T* (FT) under long‐day conditions (Tiwari et al., 2010; Valverde et al., 2004). In contrast, the downregulation by RNA silencing of either *AtmiP1b* or both *AtmiP1a/b* determined an earlier flowering as compared with wild‐type plants (Graeff et al., 2016). Consistently, also the Arabidopsis double mutant *bbx30‐2 bbx31‐2* obtained by CRISPR Cas flowered earlier compared to wild‐type (Heng et al., 2019). These phenotypes are explained by the capacity of *At*miP1a/b to interact with CO and recruit the flowering inhibitory protein TPL (Graeff et al., 2016). This model is also in agreement with the observation that *At*BBX30 and *At*BBX31 are predominantly expressed in the vasculature of leaves, similarly to CO and FT (Graeff et al., 2016).

The involvement of TCMP‐2 in the control of flowering time is corroborated by its *in vitro* interaction with *At*miP1b and by its ectopic overexpression in Arabidopsis, which results in an anticipated flowering transition and *FT* mRNA induction. The overexpression of the *TCMP‐2* gene in Arabidopsis phenocopies the effects of *At*miP1a/b silencing . Thus, although *TCMP‐2* is a Solanaceous‐specific gene, the altered flowering phenotype caused by its ectopic overexpression proved that the TCMP‐2 protein is able to interact with components of the signaling pathway controlling flowering transition in Arabidopsis, most probably thanks to its capacity to bind B‐box proteins.

**FIGURE 8:**
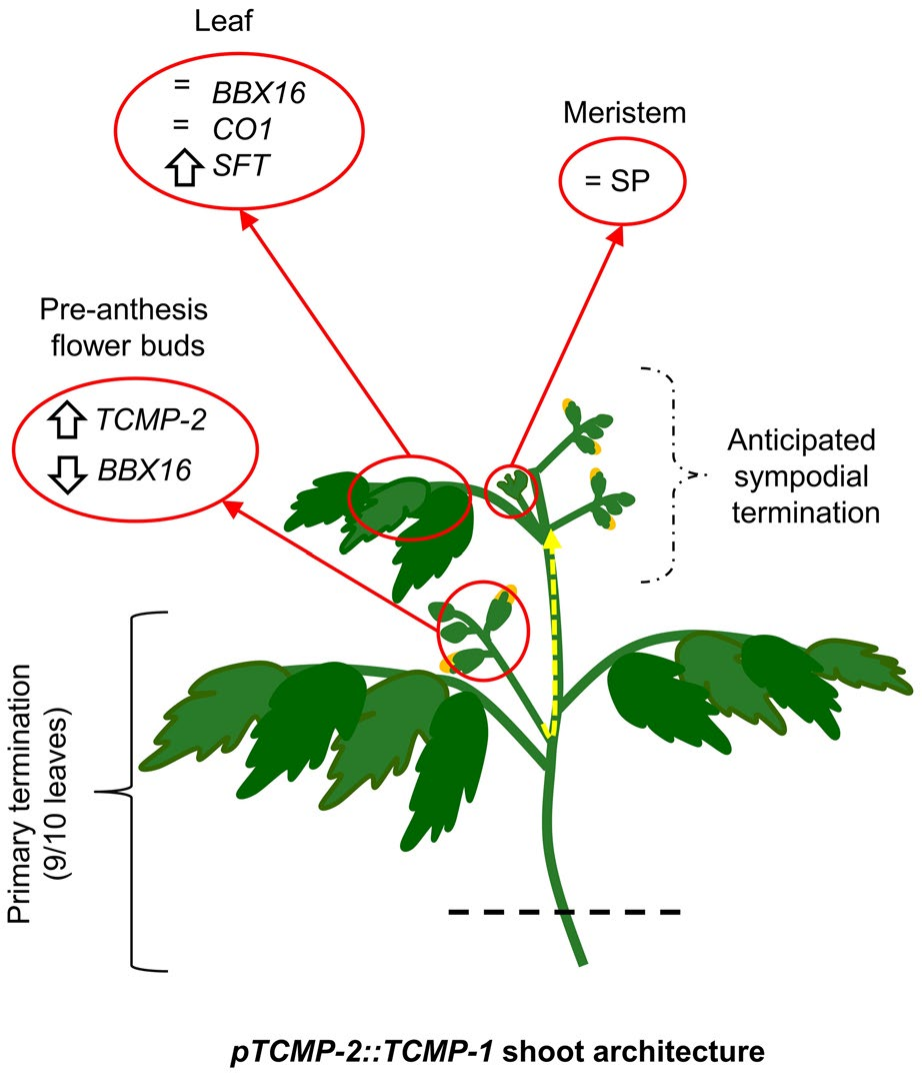
Hypothetical model of TCMP‐2 and *Sl*BBX16 action in *pTCMP‐2::TCMP‐1* plants. In preanthesis flower buds of the first inflorescence, the *TCMP‐2* transcript level increases, whereas *SlBBX16* level decreases. This determines a lesser production of the *Sl*BBX16 protein, and greater sequestration by TCMP‐2. Therefore, the *Sl*BBX16 protein available for engagement in a flowering inhibiting complex in the leaf of the sympodial unit is diminished. The expression levels of *CO1*, and *SlBBX16* are unaltered in the leaf, as well as the *SP* expression in the meristem, whereas the *SFT* level increases consistently with the lesser availability of *Sl*BBX16. In this way, the SFT/SP ratio increases, causing anticipated flowering and sympodial unit termination. The presence of *Sl*BBX16 protein in the phloem is marked by a dashed arrow

Until now, a role for tomato *Sl*BBX16 in reproductive development has never been described. However, based on our results, one could hypothesize that TCMPs/*Sl*BBX16 interaction represents a way to dynamically regulate *Sl*BBX16 and/or TCMPs activity on multimeric complexes during flower development. On these premises, we can formulate a speculative model to explain the events occurring in tomato plants overexpressing TCMP‐2 in flower buds (Figure 8). *pTCMP‐2::TCMP‐1* plants produce the primary inflorescence after 9‐10 leaves as in wild‐type plants; in these flower buds the *TCMP‐2* mRNA level is increased, whereas the *SlBBX16* mRNA level is diminished compared to the wild‐type. The interaction between TCMP‐2 and *Sl*BBX16 would further reduce the *Sl*BBX16 protein available for the formation of a putative flowering regulatory complex. In this way, the diminished level of the *Sl*BBX16 protein could result in increased *SFT* transcription in the leaves of the sympodial units. Consistently, the SFT/SP ratio would be modified in a way that favors the sympodial termination (Figure 8).

Since *Solanum lycopersicum* differs from Arabidopsis in the photoperiodic control (i.e. day‐neutral vs. long‐day), TCMP‐2 could represent an additional regulatory element, which through interaction with B‐box microProteins, modulates the effects of environmental factors (e.g. temperature) or endogenous signals on flowering regulation. Tomato is considered a day‐neutral crop, although several studies demonstrated that some cultivars have a weak photoperiodic sensitivity (Soyk et al., 2017). Notably, some wild species (e.g. *Solanum pennellii*) flower faster under short days, and it was suggested that modification in the photoperiod response occurred during domestication (Soyk et al., 2017). Intriguingly, the *S. pennellii* genome does not contain genes homologous to TCMP‐2, but only TCMP‐1 homologs (Figure S4), whereas genes homologous to both TCMPs were found in *Solanum pimpinellifolium*, which is the closest wild relative of the cultivated tomato. The photoperiodic response of *S. pimpinellifolium* displays varying degrees of day‐length sensitive flowering in different accessions (Soyk et al., 2017). Variants of TCMP‐2 genes might have been selected during the domestication process.

It would be interesting to study by reverse genetic methods the role of *Sl*BBX16 in tomato and to investigate whether an inhibitory flowering complex similar to that described in Arabidopsis (i.e. the trimeric complex miPs/CO/TPL) could also act in this species. There is another important point that needs to be addressed. As mentioned, tomato plants with increased *TCMP‐2* expression in flower buds before anthesis display an anticipated fruit production (Molesini et al., 2018). In addition, *TCMP‐2* and *SlBBX16* are co‐expressed also in fruit from the initial stages of growth to maturation. Future studies are necessary to understand the significance of the TCMP‐2/*Sl*BBX16 interaction during fruit setting and development.

### 4.1 Sequences of primers

The primers employed in this work are listed in Table S3.

## AUTHOR CONTRIBUTIONS

B.M. and T.P. conceived and designed this work; V.D., F.P., and F.M. performed the molecular analyses; G.P.D.S performed the ratiometric Bimolecular Fluorescence Complementation (rBiFC) assay; S.Z. performed the homology and docking models; A.M. and A.F. carried out the Arabidopsis transformation, G.L.R. contributed to the analysis of transgenic tomato plants; B.M. and T.P. wrote the manuscript.

## Supporting information

Fig S1‐S4

Table S1‐S3
