# Supplementary material for: TCMP‐2 affects tomato flowering and interacts with BBX16, a homolog of the arabidopsis B‐box MiP1b
Source: Plant Direct. 2020 Nov 7;4(11):e00283. doi: 10.1002/pld3.283 (PMC7648202; doi:10.1002/pld3.283)
Supplement: Supplementary file 1 — Fig S1‐S4 [file PLD3-4-e00283-s001.pdf]

Supplemental Materials

Supplemental Figure 1. Confocal images of tobacco epidermal cells agroinfiltrated with the vector containing the construct nYFP::TCMP-2//BBX16::cYFP. A, YFP complemented fluorescence (in green) can be distinguished in between cytosolic RFP (in red) and chlorophyll epifluorescence (in blue); B, after subtraction of overlapping signal, YFP fluorescence appears distributed on peripheral cellular membranes. Scale bars: 20 µm.

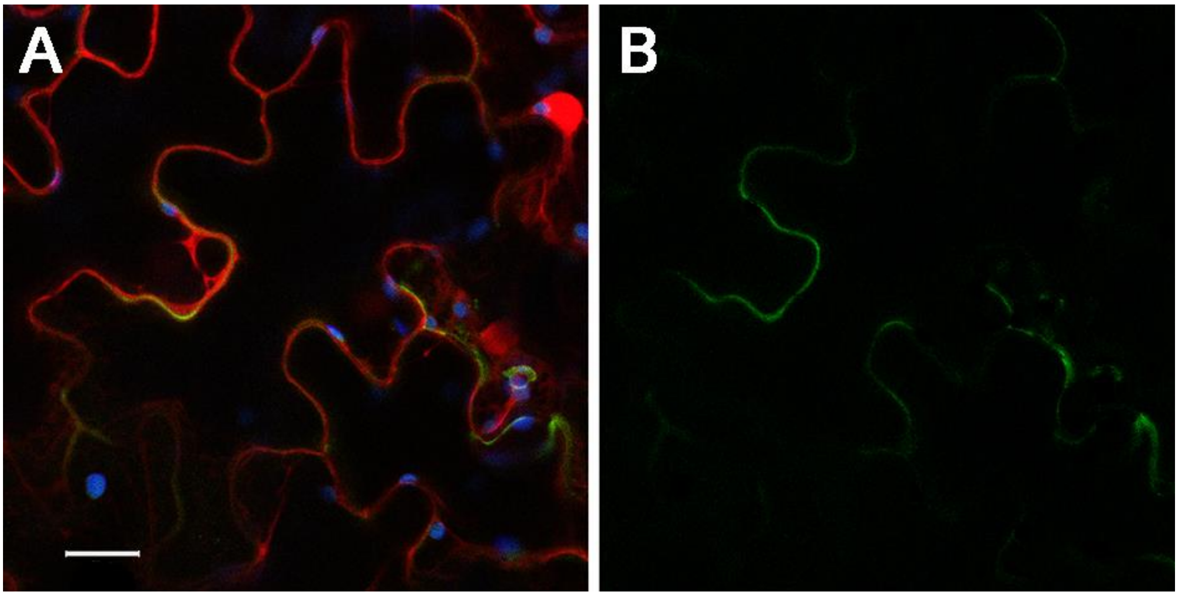

Supplemental Figure 2. Expression analysis of *35S::TCMP-2 Arabidopsis* plants. RT-PCR analysis was carried out on wild-type plants and transgenic #B2 and #M3 lines using primers pairs on actin and *TCMP-2* coding sequence. A, Agarose gel showing RT-PCR products. NTC means not template control. B, qRT-PCR analysis reporting the expression level of the transgene in the two *35S::TCMP-2* lines. The values reported are means ± SE (n= 3).

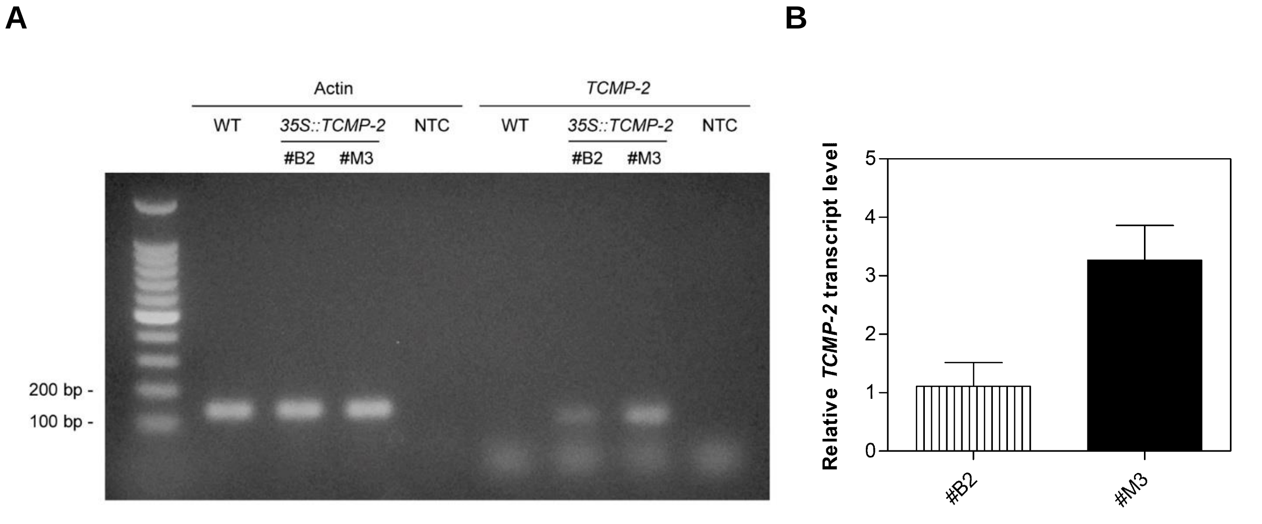

Supplemental Figure 3. Representative picture of irregular SU displayed by *pTCMP-2::TCMP-1* plants. A, Schematic diagram showing shoot architecture of a wild-type (WT) determinate tomato plant cv UC82. B, Schematic diagram showing shoot architecture of *pTCMP-2::TCMP-1* plant. The first inflorescence of the primary shoot is formed after 9/10 leaves as in wild-type. After the primary inflorescence, the shoot terminates with an irregular SU composed by a single leaf and two consecutive inflorescences.

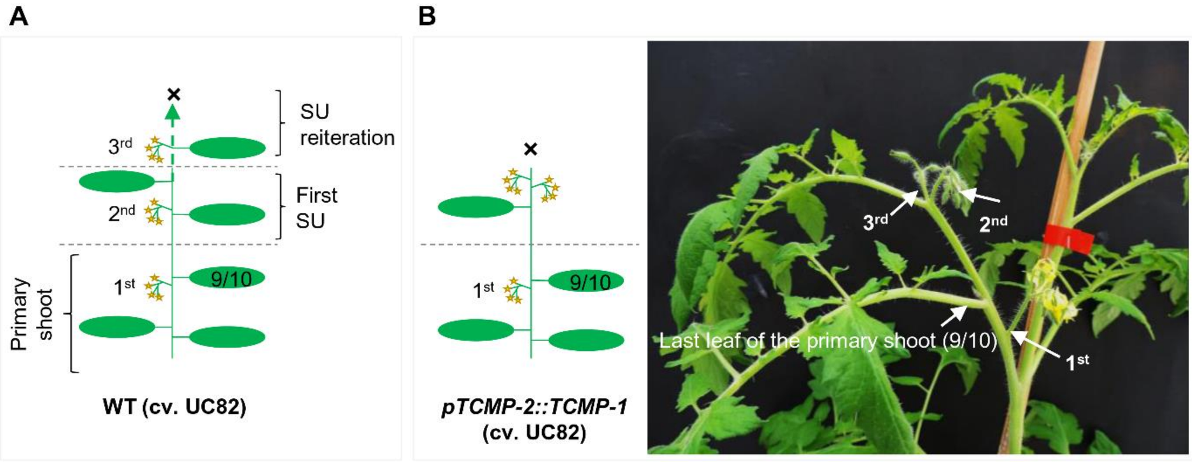

Supplemental Figure 4. Sequence alignment of TCMP-2 and TCMP-1 proteins from *Solanum lycopersicum* with the closest homologs identified in *Solanum pennellii* and *Solanum pimpinellifolium*. The alignment was performed using Multalign (Corpet, 1988). Conserved residues are coloured in red (high consensus level 90%) and in blue (low consensus level 50%). A position with no conserved residue is represented by a dot in the consensus line. The consensus symbols are ! (IV), and # (NDQE).

```
               1       10        20        30        40        50        60        70        80        90     97
               |--------+---------+---------+---------+---------+---------+---------+---------+---------+------|
     SlTCMP-2  MAAKNSEMKFAIFFVVLLTTTLVDMSGISKMQVMALRDIPPQETLLKMKLLPTNILGLCNEPCSSNSDCIGITLCQFCKEKTDQYGLTYRTCNLLP
Sopim07g049140 MAAKNSEMKFAIFFVVLLTTTLVDMSGISKMQVMALRDIPPQETLLKMKLLPTNILGLCNEPCSSNSDCIGITLCQFCKEKTDQYGLTYRTCNLLP
     SlTCMP-1       MAQKFTILFTILLV-------------VIAAQDVMAQDATL-TKLFQ-QYDPVCHKPCSTQDDCSGGTFCQACWRFAGTCGPYVGRAMAIGV
Sopim07g007250      MAQKFTILFTILLV-------------VIAAQDVMAQDATL-MKLFQ-QYDPVCHKPCSTQDDCSGGTFCQACWRFAGTCGPYVGRAMAIG
Sopen07g003310      MAQKFTILFTILLV-------------VIAAQDVMAQDATV-MKLFQ-QYDPVCNKPCSTQDDCSGGTFCEACWRFAGTCGPYVGRDMAIGV
    Consensus  .....maqKFtIlFt!LLv.............ViAaqD!maQ#atl.mKLfq.#ydpvCnkPCSt#dDCsGgTfC#aCwrfagtcGpyvgr.maig.
```
