# Supplementary material for: TCMP‐2 affects tomato flowering and interacts with BBX16, a homolog of the arabidopsis B‐box MiP1b
Source: Plant Direct. 2020 Nov 7;4(11):e00283. doi: 10.1002/pld3.283 (PMC7648202; doi:10.1002/pld3.283)
Supplement: Supplementary file 2 — Table S1‐S3 [file PLD3-4-e00283-s002.pdf]

Supplemental Table I. List of TCMP-2 interacting candidates from Y2H screen.

Locus	PBS†	Total no. of clones	No. of independent clones	Frame‡	SOL annotation
Solyc01g111600	C	6	5	IF(§)	Metal ion binding protein
Solyc02g079850	D	1		IF	Pleckstrin homology domain-containing family F member 2
Solyc03g112230	D	2	2	IF	ZZ type zinc finger domain-containing protein
Solyc06g083250	D	1		IF	Unknown Protein
Solyc12g005750	C	5	2	IF(§)	B-box Zinc finger protein CONSTANS-LIKE 4
Solyc01g008960	D	2	2	IF	Argonaute 4a
Solyc01g008280	D	1		IF	Serine/threonine-protein phosphatase 2A activator 2
Solyc01g102410	D	2	2	IF	Glutaminyl-tRNA synthetase
Solyc01G067390	D	1		IF	RNA helicase DEAH-box1
Solyc02G71980	D	1		IF	Actin-binding protein
Solyc05g056280	C	4	4	IF	RNA-binding protein Luc7-like 2
Solyc07g042190	D	1		IF	Protein of unknown function
Solyc03g123460	D	2	1	IF	Protein of unknown function
Solyc01g073890	D	1		IF	CHP-rich zinc finger protein-like
Solyc05g012610	D	1		IF	Appr-1-p processing enzyme domain protein
Solyc01g104030	D	1		IF	Inward rectifier potassium channel-like protein
Solyc09g074880	D	2	1	IF	Homology to unknown gene
Solyc06g083250	D	4	3	IF	Unknown Protein
Solyc04g015200	D	1		IF	6-phosphofructokinase 2
Solyc05g012770	D	1		IF	WRKY transcription factor 4
Solyc00g007220	D	1		IF	Ring finger protein
Solyc03g115230	D	1		IF	Heat shock protein tfhs1
Solyc02g062000	D	1		IF	RUN and FYVE domain-containing protein 1
Solyc07g021750	D	1		IF	Cytidine deaminase
Solyc08g023280	D	1		IF	Tripartite motif-containing 22
Solyc10g061930	D	1		IF	Casein kinase II subunit beta-4
Solyc05g007060	D	1		IF	Uncharacterized protein
Solyc09g075830	D	1		IF	Time for coffee
Solyc06g071450	D	1		IF(§)	RNA polymerase II transcription factor B subunit 4
Solyc07g017490	D	1		IF	Red family protein
Solyc09g007180	C	3	3	IF	Adenylate kinase
Solyc03g083000	D	1		IF	AT2G46550 protein

Solyc08g005150	D	1		IF	Ubiquitin ligase
Solyc01g008560	D	1		IF	NAD kinase 1
Solyc10g075035	D	1		IF	B3 domain-containing protein
Solyc01g110120	D	1		IF	V-type proton ATPase subunit a
Solyc03g116140	D	2	2	IF	Activating signal cointegrator 1
Solyc07g064910	D	2	1	IF	EH domain-containing protein 1
Solyc01g009780	D	2	1	IF	LITAF-domain containing protein
Solyc01g108180	D	2	1	IF	Pentatricopeptide repeat-containing protein
Solyc10g079370	D	1		IF	Transcription initiation factor IIB
Solyc04g071350	C	6	2	IF	Exocyst complex component *Sec5*
Solyc04g054760	D	1		IF	Senescence-associated family protein
Solyc06g072460	D	1		IF	Cysteine/Histidine-rich C1 domain family protein
Solyc11g066130	D	1		IF	Osmotin
Solyc07g043420	D	1		IF	2-oxoglutarate-dependent dioxygenase 2
Solyc09g008280	D	1		IF	S-adenosyl-L-methionine synthetase

†PBS (predicted biological score) shows the confidence of interaction: C, good; D, moderate.

‡Frame (IF:*In frame* with the Gal4 Activation Domain). (§), the fragment contains the full length ORF

Supplemental Table II. List of protein sequences analysed using MEGA5 for the phylogenetic tree construction reported in Figure 3.

Arabidopsis thaliana

Domains	Locus (gene name)	Protein sequence
1 B-box + CCT	At1g25440 (*At*BBX15-COL16)	MMKSLANAVGAKTARACDSCVKRRARWYCAADDAFLCQSCDSLVHSANPLARRHERVRLKTASPAVVKHSNHSSASPPH EVATWHHGFTRKARTPRGSGKKNNSSIFHDLVPDISIEDQTDNYELEEQLICQVPVLDPLVSEQFLNDVVEPKIEFPMIRSGL MIEEEEDNAESCLNGFFPTDMELEEFAADVETLLGRGLDTESYAMEELGLSNSEMFKIEKDEIEEEVEEIKAMSMDIFDDDRK DVDGTVPFELSFDYESSHKTSEEEVMKNVESSGECVVKVKEEEHKNVLMLRLNYDSVISTWGGQGPPWSSGEPPERDMDI SGWPAFSMVENGGESTHQKQYVGGCLPSSGFGDGGREARVSRYREKRRTRLFSKKIRYEVRKLNAEKRPRMKGRFVKRA SLAAAASPLGVNY
1 B-box + CCT	At1g68520 (*At*BBX14-COL6)	MMKSLASAVGGKTARACDSCVKRRARWYCAADDAFLCHACDGSVHSANPLARRHERVRLKSASAGKYRHASPPHQATW HQGFTRKARTPRGGKKSHTMVFHDLVPEMSTEDQAESYEVEEQLIFEVPVMNSMVEEQCFNQSLEKQNEFPMMPLSFKSS DEEDDDNAESCLNGLFPTDMELAQFTADVETLLGGGDREFHSIEELGLGEMLKIEKEEVEEEGVVTREVHDQDEGDETSPF EISFDYEYTHKTTFDEGEEDEKEDVMKNVMEMGVNEMSGGIKEEKKEKALMLRLDYESVISTWGGQGIPWTARVPSEIDLD MVCFPTHTMGESGAEAHHHNHFRGLGLHLGDAGDGGREARVSRYREKRRTRLFSKKIRYEVRKLNAEKRPRMKGRFVKR SSIGVAH
2 B-box	At2g21320 (*At*BBX18-DBB1a)	MRILCDACESAAAIVFCAADEAALCCSCDEKVHKCNKLASRHLRVGLADPSNAPSCDICENAPAFFYCEIDGSSLCLQCDMV VHVGGKRTHRRFLLLRQRIEFPGDKPNHADQLGLRCQKASSGRGQESNGNGDHDHNMIDLNSNPQRVHEPGSHNQEEGI DVNNANNHEHE
2 B-box + CCT	At3g02380 (*At*BBX3-COL2)	MLKEESNESGTWARACDTCRSAACTVYCEADSAYLCTTCDARVHAANRVASRHERVRVCQSCESAPAAFLCKADAASLCT ACDAEIHSANPLARRHQRVPILPLSANSCSSMAPSETDADNDEDDREVASWLLPNPGKNIGNQNNGFLFGVEYLDLVDYSS SMDNQFEDNQYTHYQRSFGGDGVVPLQVEESTSHLQQSQQNFQLGINYGFSSGAHYNNNSLKDLNHSASVSSMDISVVPE STASDITVQHPRTTKETIDQLSGPPTQVVQQLTPMEREARVLRYREKKKTRKFDKTIRYASRKAYAEIRPRIKGRFAKRIETEA EAEEIFSTSLMSETGYGIVPSF
2 B-box + CCT	At3g07650 (*At*BBX7-COL9)	MGYMCDFCGEQRSMVYCRSDAACLCLSCDRSVHSANALSKRHSRTLVCERCNAQPATVRCVEERVSLCQNCDWSGHNN SNNNNSSSSSTSPQQHKRQTISCYSGCPSSSELASIWSFCLDLAGQSICEQELGMMNIDDDGPTDKKTCNEDKKDVLVGSS SIPETSSVPQGKSSSAKDVGMCEDDFYGNLGMDEVDMALENYEELFGTAFNPSEELFGHGGIDSLFHKHQTAPEGGNSVQ PAGSNDSFMSSKTEPIICFASKPAHSNISFSGVTGESSAGDFQECGASSSIQLSGEPPWYPPTLQDNNACSHSVTRNNAVM RYKEKKKARKFDKRVRYASRKARADVRRRVKGRFVKAGEAYDYDPLTPTRSY

1 B-box	At3g21890 (*At*BBX30-MIP1a)	MCRGLNNEESRRSDGGGCRSLCTRPSVPVRCELCDGDASVFCEADSAFLCRKCDRWVHGANFLAWRHVRRVLCTSCQK LTRRCLVGDHDFHVVLPSVTTVGETTVENRSEQDNHEVPFVFL
1 B-box	At4g15248 (*At*BBX31-MIP1b)	MCRGFEKEEERRSDNGGCQRLCTESHKAPVSCELCGENATVYCEADAAFLCRKCDRWVHSANFLARRHLRRVICTTCRKL TRRCLVGDNFNVVLPEIRMIARIEEHSSDHKIPFVFL
2 B-box	At4g38960 (*At*BBX19-DBB1b)	MRILCDACENAAAIIFCAADEAALCRPCDEKALHMRLDISKCSESVKRVQIVETSSLIWWIKMGTFCLQSLHLVVHMCNKLAS RHVRVGLAEPSNAPCCDICENAPAFFYCEIDGSSLCLQCDMVVHVGGKRTHGRFLLLRQRIEFPGDKPKENNTRDNLQNQR VSTNGNGEANGKIDDEMIDLNANPQRVHEPSSNNNGIDVNNENNHEPAGLVPVGPFKRESEK
2 B-box + CCT	At5g15840 (*At*BBX1-CO)	MLKQESNDIGSGENNRARPCDTCRSNACTVYCHADSAYLCMSCDAQVHSANRVASRHKRVRVCESCERAPAAFLCEADD ASLCTACDSEVHSANPLARRHQRVPILPISGNSFSSMTTTHHQSEKTMTDPEKRLVVDQEEGEEGDKDAKEVASWLFPNSD KNNNNQNNGLLFSDEYLNLVDYNSSMDYKFTGEYSQHQQNCSVPQTSYGGDRVVPLKLEESRGHQCHNQQNFQFNIKYG SSGTHYNDNGSINHNAYISSMETGVVPESTACVTTASHPRTPKGTVEQQPDPASQMITVTQLSPMDREARVLRYREKRKTR KFEKTIRYASRKAYAEIRPRVNGRFAKREIEAEEQGFNTMLMYNTGYGIVPSF
2 B-box + CCT	At5g15850 (*At*BBX2-COL1)	MLKVESNWAQACDTCRSAACTVYCRADSAYLCSSCDAQVHAANRLASRHERVRVCQSCERAPAAFFCKADAASLCTTCD SEIHSANPLARRHQRVPILPISEYSYSSTATNHSCETTVTDPENRLVLGQEEEDEDEAEAASWLLPNSGKNSGNNNGFSIGD EFLNLVDYSSSDKQFTDQSNQYQLDCNVPQRSYGEDGVVPLQIEVSKGMYQEQQNFQLSINCGSWGALRSSNGSLSHMV NVSSMDLGVVPESTTSDATVSNPRSPKAVTDQPPYPPAQMLSPRDREARVLRYREKKKMRKFEKTIRYASRKAYAEKRPRI KGRFAKKKDVDEEANQAFSTMITFDTGYGIVPSF
2 B-box + CCT	At5g48250 (*At*BBX8-COL10)	MGYMCDFCGEQRSMVYCRSDAACLCLSCDRNVHSANALSKRHSRTLVCERCNAQPASVRCSDERVSLCQNCDWSGHDG KNSTTTSHHKRQTINCYSGCPSSAELSSIWSFCMDLNISSAEESACEQGMGLMTIDEDGTGEKSGVQKINVEQPETSSAAQ GMDHSSVPENSSMAKELGVCEDDFNGNLISDEVDLALENYEELFGSAFNSSRYLFEHGGIGSLFEKDEAHEGSMQQPALSN NASADSFMTCRTEPIICYSSKPAHSNISFSGITGESNAGDFQDCGASSMKQLSREPQPWCHPTAQDIIASSHATTRNNAVMR YKEKKKARKFDKRVRYVSRKERADVRRRVKGRFVKSGEAYDYDPMSPTRSY

Solanum lycopersicum

**Domains**	**Locus (gene name)**	**Protein sequence**
1 B-box	Solyc12g005750 (*Sl*BBX16)	MCNGRREIDEEKIEELHNIIVCELCKSEAYVYCEADNAFLCKKCDKLVHTANFFAQRHIRCILCGICKKLTKRYLIGVSHEVILLKVVRCTNFDEQNCSTKVKEPFLFL
1 B-box	Solyc07g052620 (*Sl*BBX17)	MCSGRREGDEKTSSTSYCKGPSKEGESIISSAITCALCSSEASVYCEADNAFLCRKCDRSVHGANFLAQRHIRCLLCSVCRKTTRRFLIGTSSELILPTIARLEQRNRSRSAESETTDYRTTPQELFLFI
2 B-box	Solyc12g089240 (*Sl*BBX20)	MKIQCDVCNKKEAIVFCTADEAALCDDCDHRVHHVNKLASKHQRFSLVQPSPKQAPMCDICQERRGFLFCQQDRAIMCRECDIPIHKANEHTQKHNRYLLTGIKLSANSALYSAPSQSQSQSQSAISSADSCVSNLKSKDSTSKPVAGSVFVSPAIISNSTKGGAVSSAVESVKVVKEKVGGCNNNVQFVNGGGNNLTSSISEYLEMLPGWHVEDFLDCSTPNVYSKNIGDEDMLSFWDTDLESQFSSFPPQNVGIWVPQAPPLQESKQETQIQFFPSQNLNFGGQIGLKESREVTNIKSSRKWTDDNSFAVPQMKPPSTSFKRTRTLW
2 B-box	Solyc04g081020 (*Sl*BBX21)	MKIQCDVCNNNEASVFCVADEAALCDSCDHRVHHANKLASKHQRFSLIQPSPKQIPVCDICQERRAFLFCQQDRAILCRECDVSIHKANEHTQKHNRFLLTGVKISANSSLYTSSESVSAASCSANQDSVTNLNKPQICTKKTSPVSGSVPQQQVSVAANIGENSYTSSISEYLEMLPGWHVEELLNASTIPTNGFCKIGDNDVFPIWDSEIESSMNSFSPENIGIWVPQAPPALTPQKNQNQVFPRNINFGGQIEFKNMKEVTSKKSSRKWRDDNSFAVPQISPSSSSISFKRSRTLW
1 B-box + CCT	Solyc05g009310 (*Sl*BBX15)	MVSERKLASAMGGKTTRACDNCIKKRARWYCPADDAFLCQNCDASVHSANPLARRHERVRLKTSSLKQTSSPSSSSDDYFPDLESPLSISSVSVSVSVPSWHRGFTRKARTPRQGRKASKSAGDGDVIRKNPIHLVPEILSDENSLDENEEEQLLYRVPILDPFVGHLYSSSTAPTDADSEFKLESKEMTLQDDICNVDLNRFHEMLPSEMELAEFAADVESLLGKGLDDESFDMEGLGLLGVCNKEENSMISHEKVKIEDEGEMEVVTKTTSPTTHNHQYNHTHDHDIDINEDTFEFKFDYDSSINIIGDDEVVTNDENKKKILLNLDYEGVLKAWADQRCPWTNGERPELDSNESWPDCMGNYMGIMNENVTIVDRGREARVTRYREKRRTRLFSKKIRYEVRKLNAEKRPRMKGRFVKRANFVTTSTPNYPLVK
1 B-box + CCT	Solyc04g007210 (*Sl*BBX13)	MSSEKKLANAMGAKTARACDNCIRKRARWYCAADDAFLCQSCDSSVHSANPLARRHERVRLKTSSFKSSDDFPNLESTVSGLGSGSGSGSDSIPSWHCGFTRKARTPRYGNKHAKRVKSTEEEEEEEEMKNPIQLVVPEILSDENSHDENEEEQLLYRVPIFDPFMADGSNYGNEYSSNKVDFNQDMNTFQGLLAPSEMELAEFAADVVSLLGKGLDDEESFNYMEGLGFLEKHDEKLVKVEDEGEVGFVNMISTNNQVDYSEFDMVGETFELKFDYDSQVINNLDEDNKKVEFLEINYDSGKNNNKIMLNLDYESVLKSWGDKRFPWTTGVRPEVDFNDCWPVCMGNCGKIHSYGDIAIMNGHGGGVVDEGREARVLRYKEKRRTRLFSKKIRYEVRKLNAEKRPRMKGRFVKRTNFAPTPFPSLNK

2 B-box + CCT	Solyc02g089540 (*Sl*BBX3)	MLKKENSNNWARVCDSCHSATCTVYCRADSAYLCAGCDARIHTASLMASRHERVWVCEACERAPAAFLCKADAASLCASC DADIHSANPLARRHHRVPIMPIPGTIYGPPAVHTITGGSMMIGGTTGEGTEDDGFLSLNQDADDTTIDEEDEDEAASWLLLNP PVKNNNKNNNYGMLFGGEVVDDYLDLAEYGGDSQFNDQYSVNQQQQHYSVPQKSYVEDSVVPVQNGQRKSLILYQTPQQ QQSHHLNFQLGMEYDNSNTGYGYPASLSHSVSISSMDVSVVPESAQSETSNSHPRPPKGTIDLFSGPPIQIPPQLTPMDREA RVLRYREKKKNRKFEKTIRYASRKAYAETRPRIKGRFAKRTDVEAEVDQMFSTQLMTDSNYGIVPSF
2 B-box + CCT	Solyc12g096500 (*Sl*BBX5)	MGTENWSLTAKLCDSCKTSPATVFCRADSAFLCLGCDCKIHAANKLASRHARVWVCEVCEQAPASVTCKADAAALCVTCD RDIHSANPLARRHERFPVVPFYDFAVAKSHGGGDTDADAVDDEKYFDSTNENPSQPEEEAEAASWILPTPKEGTDQYKSAD YLFNDMDSYLDIDLMSCEQKPHILHHQQHQHNHYSSDGVVPVQNNNETTHLPGPVVDGFPTYELDFTGSKPYMYNFTSQSI SQSVSSSSLDVGVVPDHSTMTDVSNTFVMNSSSGAIAGAGADVVPNAVSGLDREARVMRYREKRKNRKFEKTIRYASRKA YAETRPRIKGRFAKRTETEIDSLITVDASYGVVPSF

Supplemental Table III. Sequences of the primers.

Name	Experiment	Forward Sequence (5‘ → 3‘)	Reverse Sequence (5‘ → 3‘)
SlSP *Solyc06g074350*	qRT-PCR	TTAGTGCGTGGACGGACTACTACT	GAGGGTGAACAACCATTAACCCCC
SlSFT *Solyc03g063100*	qRT-PCR	AAACAGTGTATGCTCCAGGATGGC	AGATCTTCTACGTCCACCACTGCC
SlCO1 *Solyc02g089540*	qRT-PCR	AAGATCAACCACAAGCCCCTAA	TCCTCTGTGGAAAGCTGCTGTAG
SlActin *Solyc11g005330*	qRT-PCR	TTCAAAGGGCGAGTACGACGAG	CAGCAGACCCGAGTTCACTTTT
SlBBX16 *Solyc12g005750*	qRT-PCR	TGTGAACTTTGTAAATCAGAAGCCT	TGCCTTTGAGCAAAGAAATTAGC
SlTCMP-2 *Solyc07g049140*	qRT-PCR	ACGAACCTTGCAGCTCAAAC	GCAACAGGTTGCATGTACGG
AtFT *At1g65480*	qRT-PCR	ACTGCAGGAATTCATCGTGTCGTG	GCAGCCACTCTCCCTCTGACAATT
AtActin *At3g18780*	qRT-PCR	TGTTCTCTCCTTGTACGCCAGT	CAGCAAGGTCAAGACGGAGGA
***Sl*BBX16** Solyc12g005750	Y2H	GGAGGCCAGTGAATTCTGCAATGGAAGAAGAGAAATTGA	CGAGCTCGATGGATCCGAGAAACAAAAAAGGTTCTTTC
***Sl*TCMP-2** Solyc07g049140	Y2H	CATGGAGGCCGAATTCACAAATATTTTGGGACTTTGTAACG	GCAGGTCGACGGATCCAGGCAACAGGTTGCATGTACG
***At*miP1b** At4g15248	Y2H	GGAGGCCAGTGAATTCTGTAGAGGGTTTGAGAAAGAAGAAG	CGAGCTCGATGGATCCGAGAAACACAAAGGGAATTTTGTG
***Sl*CO1** Solyc02g089540	Y2H	GGAGGCCAGTGAATTCTTGAAAAAAGAGAACAGTAACAATTGGG	CGAGCTCGATGGATCCGAATGAAGGGACAATTCCATAATTGC
***Sl*TPL1** Solyc03g117360	Y2H	GGAGGCCAGTGAATTCTCATCTCTCAGTAGAGAGCTTG	CGAGCTCGATGGATCCTCTTGGTGCTTGATCGGAGC
p35S::TCMP-2::tNOS	Transgenic state of *35S::TCMP-2 Arabidopsis* plants	CTTCGTCAACATGGTGGAGCACGACA	GATCTAGTAACATAGATGACACCG

pTCMP-2::TCMP-1	Transgenic state of *pTCMP-2::TCMP-1* tomato plants	CTCGAGCCCTTTAAAAAGTAT	TTATCACACGCCTATGGCCATGGC
nYFP::TCMP-2 //BBX16::cYFP	Ratiometric BIFC	TCMP-2 cloning<br>GGGGACAACTTTGTATAATAAAGTTGCCATGACAAATATTTTGGGACTTTGTAA<br>BBX16 cloning<br>GGGGACAAGTTTGTACAAAAAAGCAGGCTTTATGTGCAATGGAAGAAGAGAAA	TCMP-2 cloning (with Stop codon)<br>GGGGACCACTTTGTACAAGAAAGCTGGGTATTAAGGCAACAGGTTGCATGTAC<br>BBX16 cloning (without Stop codon)<br>GGGGACAACTTTGTATAGAAAAGTTGGGTGAGAAACAAAAAAGGTTCTTTCA
**Name**	**Experiment**	**Forward Sequence (5‘ → 3‘)**	**Reverse Sequence (5‘ → 3‘)**
TCMP-2::nYFP //BBX16::cYFP	Ratiometric BIFC	TCMP-2 cloning<br>GGGGACAACTTTGTATAATAAAGTTGCCATGACAAATATTTTGGGACTTTGTAA<br>BBX16 cloning<br>GGGGACAAGTTTGTACAAAAAAGCAGGCTTTATGTGCAATGGAAGAAGAGAAA	TCMP-2 cloning (without Stop codon)<br>GGGGACCACTTTGTACAAGAAAGCTGGGTAAGGCAACAGGTTGCATGTAC<br>BBX16 cloning (without Stop codon)<br>GGGGACAACTTTGTATAGAAAAGTTGGGTGAGAAACAAAAAAGGTTCTTTCA
nYFP::TCMP-2 //ΔBBX16::cYFP	Ratiometric BIFC	TCMP-2 cloning<br>GGGGACAACTTTGTATAATAAAGTTGCCATGACAAATATTTTGGGACTTTGTAA<br>Deleted BBX16 cloning<br>GGGGACAAGTTTGTACAAAAAAGCAGGCTTTATGTGTATTCTTTGTGGAATTTGC	TCMP-2 cloning (with Stop codon)<br>GGGGACCACTTTGTACAAGAAAGCTGGGTATTAAGGCAACAGGTTGCATGTAC<br>Deleted BBX16 cloning (without Stop codon)<br>GGGGACAACTTTGTATAGAAAAGTTGGGTGAGAAACAAAAAAGGTTCTTTCA
TCMP-2::nYFP //ΔBBX16::cYFP	Ratiometric BIFC	TCMP-2 cloning<br>GGGGACAACTTTGTATAATAAAGTTGCCATGACAAATATTTTGGGACTTTGTAA<br>Deleted BBX16 cloning<br>GGGGACAAGTTTGTACAAAAAAGCAGGCTTTATGTGTATTCTTTGTGGAATTTGC	TCMP-2 cloning (without Stop codon)<br>GGGGACCACTTTGTACAAGAAAGCTGGGTAAGGCAACAGGTTGCATGTAC<br>Deleted BBX16 cloning (without Stop codon)<br>GGGGACAACTTTGTATAGAAAAGTTGGGTGAGAAACAAAAAAGGTTCTTTCA
